# Expression and interaction of AGPase subunits reveal functional enzyme complexes in barley

**DOI:** 10.3389/fpls.2025.1671162

**Published:** 2025-10-16

**Authors:** Zhenbin Cheng, Boai Xi, Yan Gao, Xudong He, Jianhao Gao, Haonan Tang, Yajie Liu, Hui Zhao, Zongyun Feng, Guowu Yu

**Affiliations:** ^1^ College of Agronomy, Sichuan Agricultural University, Chengdu, China; ^2^ State Key Laboratory of Crop Gene Exploration and Utilization in Southwest China, Sichuan Agricultural University, Chengdu, China

**Keywords:** barley, grain filling, enzyme complex, starch biosynthesis, subunit assembly

## Abstract

In the starch biosynthetic pathway of Poaceae plants, ADP-glucose pyrophosphorylase (AGPase) serves as the rate-limiting enzyme that catalyzes the conversion of glucose-1-phosphate (G1P) and ATP to ADP-glucose, the immediate precursor for starch synthesis. Despite its fundamental role, the molecular characteristics and regulation of AGPase in barley (*Hordeum vulgare* L.) remain poorly understood. This study systematically investigated the expression dynamics during barley grain development and subunit interactions of AGPase in vitro. Our findings revealed distinct spatiotemporal expression patterns among AGPase, with preferential accumulation during late grain-filling stages. Co-immunoprecipitation coupled with mass spectrometry (Co-IP/MS) demonstrated specific physical interactions between small (AGPS) and large (AGPL) subunits, confirming the heterotetrameric architecture of functional AGPase complexes in barley. Enzymatic characterization showed that particular subunit combinations (AGPS1-AGPL1 and AGPS2b-AGPL2) exhibited significantly higher catalytic activity compared to other permutations. These results demonstrate that AGPase expression is developmentally regulated, specific inter-subunit interactions determine enzymatic efficiency, and optimal activity requires precise stoichiometric assembly. The demonstrated spatiotemporal coordination of AGPase subunits provides mechanistic insight into the control of starch biosynthesis during the late stage of grain filling. These results also provide a potential key target to improve barley starch synthesis and metabolism.

## 1 Introduction

Barley (*Hordeum vulgare* L.) is a member of the Poaceae family and is the fourth most widely cultivated cereal crop globally, following wheat, rice, and maize in production area (Lukinac and Jukić, 2022). Starch accounts for 55-65% of the dry weight in barley grains and is its predominant storage carbohydrate (Jeon et al., 2010; Sahoo et al., 2023). Starch also serves as the primary feedstock for industrial production of native starch, modified starch derivatives, and glucose syrups (Zarski et al., 2024).

The biochemical pathway of starch synthesis in cereal endosperm involves coordinated action of several key enzymes, including ADP-glucose pyrophosphorylase (AGPase), granule-bound starch synthase (GBSS), soluble starch synthases (SS), starch branching enzymes (SBE), debranching enzymes (DBE), and disproportionating enzymes (DPE) (Figueroa et al., 2022; Ballicora et al., 2003).

Among these, AGPase occupies a central position as it catalyzes the rate-limiting conversion of glucose-1-phosphate (G1P) and ATP to ADP-glucose, the essential glucosyl donor for starch biosynthesis (Sweetlove et al., 1999; Sun et al., 2020). Plant AGPases typically exist as heterotetrameric complexes composed of two large (AGP-L, ~50–55 kDa) and two small (AGP-S, ~51–54 kDa) subunits (Tuncel et al., 2014; Thorbjørnsen et al., 1996), with emerging evidence suggesting distinct functional specialization between the subunits. The small subunit (α_2_) contains the catalytic core and allosteric regulatory sites (Danny et al., 1999; Yu et al., 2023c). The large subunit (β_2_) modulates enzyme activity and stability (Huang et al., 2014; Kumar et al., 2024). Their interaction determines grain yield potential (Hannah et al., 2012; Kang et al., 2013).

Phylogenetic analyses reveal that AGPase subunits evolved from a common ancestral gene, maintaining high sequence conservation while acquiring specialized functions (Maharana et al., 2024; Prathap and Tyagi, 2020). This evolutionary conservation reflects the critical role of enzymes in starch metabolism, while tissue-specific expression patterns and multiple alternative subunits combinations also fine-tune enzyme activity (Batra et al., 2017; Georgelis et al., 2007). In maize (*Zea mays*), for instance, distinct heterotetrameric assemblies (SH2/BT2, SH2/LeAFs, EMB5/EMBL) exhibit different kinetic properties and regulatory responses (Sandrine et al., 2020; Yoon et al., 2021), demonstrating how combinatorial flexibility enables plants to adapt starch biosynthesis to developmental and environmental requirements (Saripalli and Gupta, 2015).

Despite extensive characterization of AGPase in model cereals, fundamental gaps remain in understanding barley AGPase, particularly regarding the structural determinants of subunit association, the molecular basis for combinatorial regulation, and functional consequences of specific subunit pairings (Yang et al., 2024; Wang et al., 2023). This study addresses these knowledge gaps through a comprehensive analysis of barley AGPase subunits, with particular focus on their developmental expression profiles, interaction networks, and biochemical characterization of different subunit combinations. Our results advance understanding of the molecular mechanisms governing starch biosynthesis in barley, with potential applications for crop improvement through targeted manipulation of AGPase subunit cooperativity.

## 2 Results

### 2.1 Temporal dynamics of AGPase activity and starch accumulation during grain development

**Figure 1:**
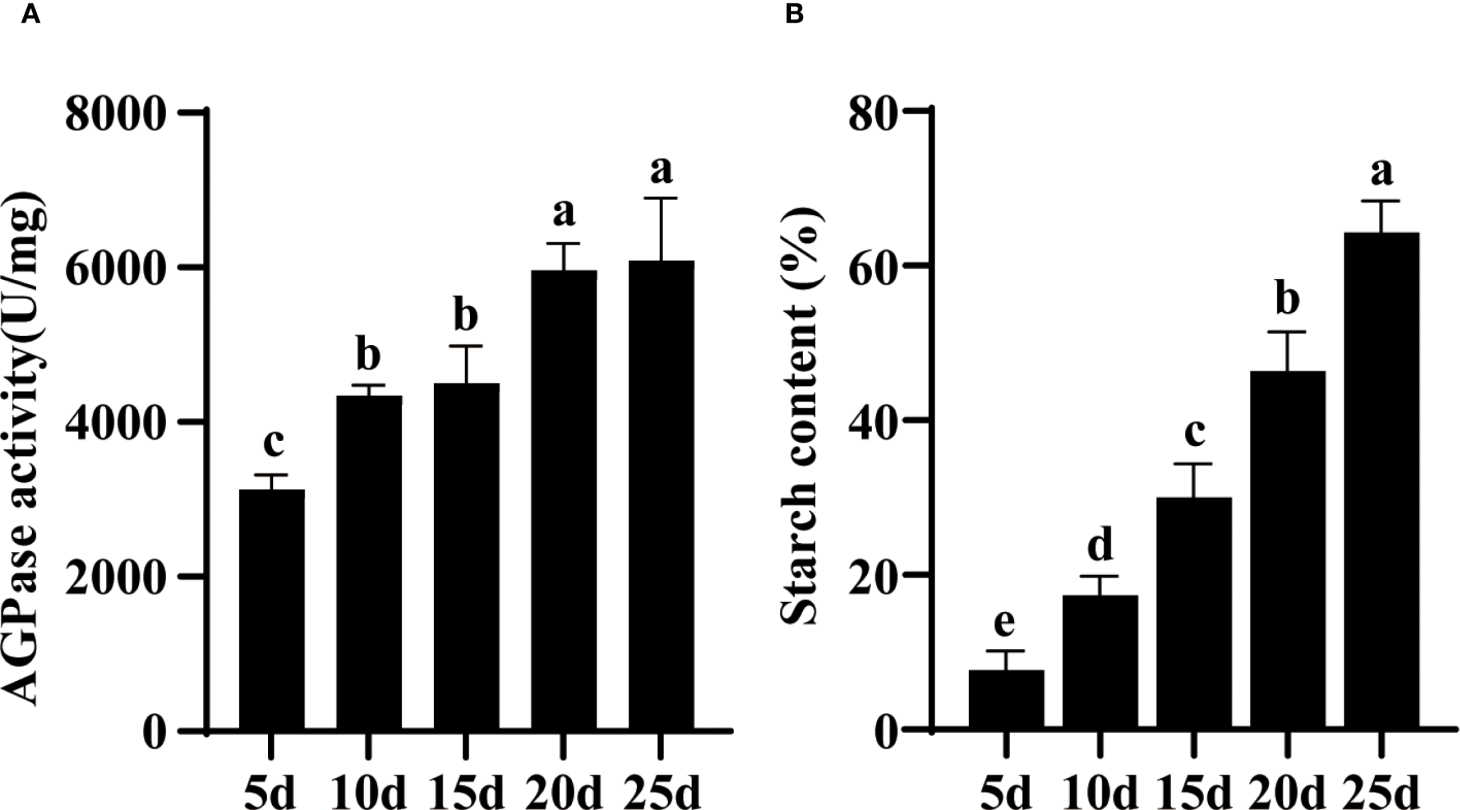
Developmental profiles of AGPase activity and starch accumulation in barley grains. **(A)** Temporal changes in AGPase activity during grain development (5–25 days after anthesis, DAA). **(B)** Corresponding starch accumulation patterns during grain development. Values represent mean ± SD of three biological replicates (n=3). Lowercase letters denote statistically significant differences (one-way ANOVA with Tukey’s *post-hoc* test, *p* < 0.05).

The temporal dynamics analysis of both have revealed similar trends in these two parameters, which AGPase activity and starch content both increase continuously with grain development (**Figures 1A, B**). The observed temporal coupling between AGPase activity and subsequent starch accumulation implies a precursor-product relationship. Meanwhile, the correlative analysis results also showed a high degree of correlation between AGPase activity and starch accumulation parameters. (**Supplementary Figure 1**).

### 2.2 Bioinformatics analysis of AGPase subunits

**Figure 2:**
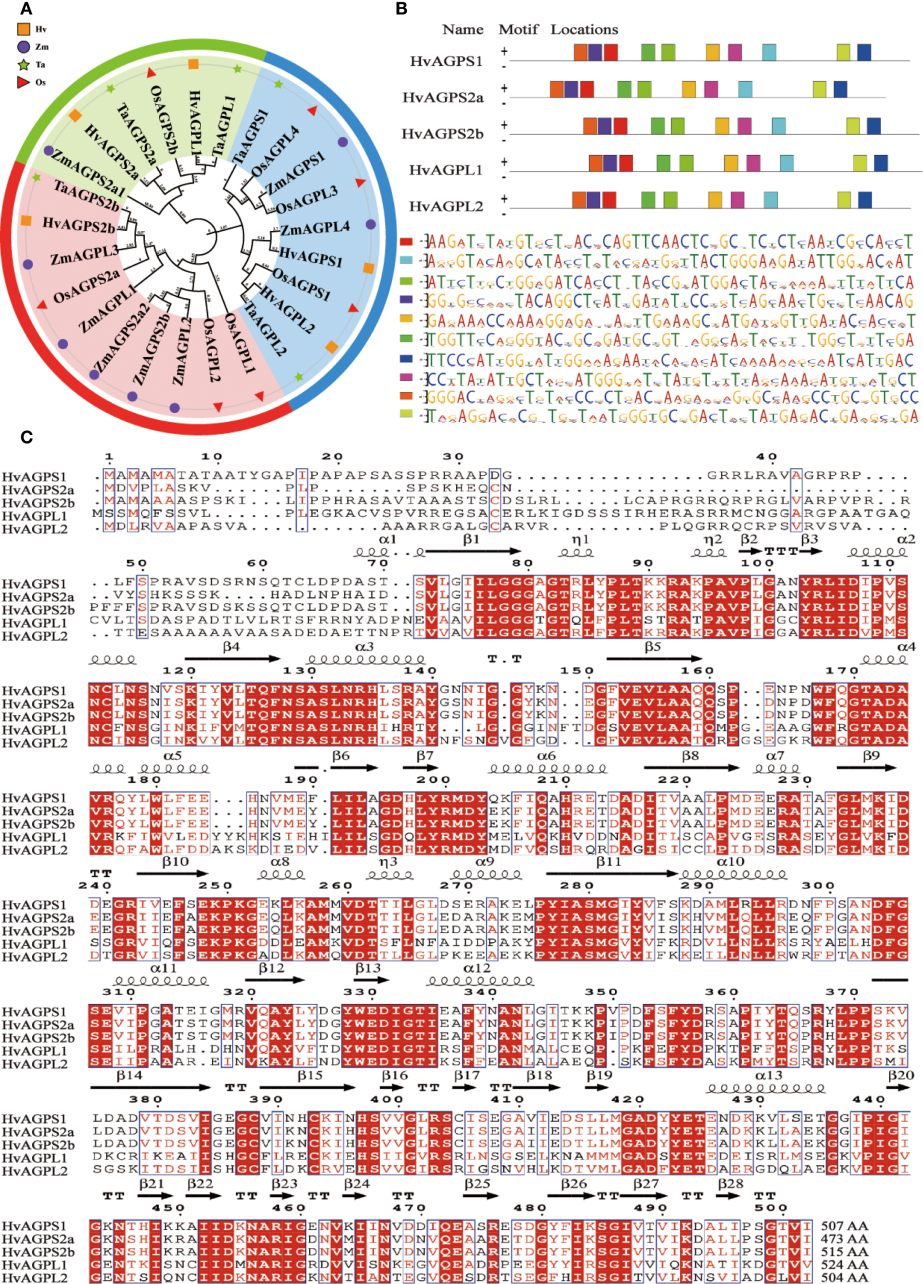
Bioinformatics characterization of barley AGPase subunits. **(A)** Phylogenetic analysis of AGPase subunits from major cereal crops. Species abbreviations: *Hv* (*Hordeum vulgare*, barley), *Ta* (*Triticum aestivum*, wheat), *Zm* (*Zea mays*, maize), *Os* (*Oryza sativa*, rice). **(B)** Conserved motif architecture of barley AGPase subunits. **(C)** Multiple sequence alignment of deduced amino acid sequences for all barley AGPase subunits. Secondary structure elements are annotated: α (alpha-helix), β (beta-sheet), TT (turn), η (η-bridge), AA(amino acid).

According to Huang’ report, the complete set of AGPase subunit genes from barley and related Poaceae species was systematically identified and retrieved from the NCBI database (**Supplementary Table 1**) (Huang et al., 2021). This comprehensive dataset included three small subunit genes (*HvAGPS1*, *HvAGPS2a*, *HvAGPS2b*) and two large subunit genes (*HvAGPL1*, *HvAGPL2*), which were successfully amplified from barley cDNA using reference sequences obtained from the barley genome database. Sequence verification through multiple alignment analysis confirmed 100% identity between all cloned sequences and their corresponding reference genes, validating the fidelity of our cloning procedures. Phylogenetic reconstruction using MEGA-X software revealed distinct evolutionary relationships among AGPase subunits from diverse Poaceae species (**Figure 2A**). The analysis demonstrated that barley AGPase subunits cluster most closely with their wheat orthologs, forming a well-supported clade within the Triticeae lineage. This close phylogenetic relationship was consistently observed for both small and large subunits. Protein motif analysis predicted conserved structural domains across all barley AGPase subunits. We can easily find that they all contain ten evolutionarily conserved Motif Locations by structural mapping the Motif Locations of different subunits, and the spatial arrangement and linear dimensions of these Motif Locations have remaining invariant. (**Figure 2B**). While the core sequence motifs were maintained between subunit, their spatial arrangement exhibited subunit-specific variations. The small subunits protein (HvAGPS1, HvAGPS2a, HvAGPS2b) shared identical motif organizations, whereas the large subunits protein (HvAGPL1, HvAGPL2) displayed distinct but conserved motif patterns. Multiple sequence alignment of the deduced amino acid sequences revealed substantial sequence conservation among barley AGPase subunits (**Figure 2C**). The alignment showed 68-72% sequence identity between small and large subunits, with particularly high conservation in regions corresponding to known functional domains. This high degree of sequence homology supports the hypothesis of common ancestral origin for both subunit types, while the observed variations likely contribute to their functional specialization. From the perspective of protein structure, in the enzyme complex of HvAGPase, its small subunit proteins are primarily responsible for catalytic function, containing binding sites for substrates (G-1-P, ATP) and the catalytic centers. Its catalytic core is similar to many sugar-nucleotidyltransferases, which belongs to the glycosyltransferase superfamily. The ATP binding site of AGPase contains a classic sugar nucleotide-binding motif, namely a Rossmann fold (β-α-β-α-β) domain, corresponding to the β7-α6-β8-α7-β9 structure (**Figure 2C**). The G-1-P binding site is located near the catalytic center and adjacent to the terminal phosphate group of ATP. Typically involving some conservative residues such as arginine, histidine, and asparagine, they recognize and bind substrates by forming a hydrogen-bond network with the phosphate groups and glucosyl moiety of G-1-P. Its large subunit proteins are mainly responsible for allosteric regulation and the primary binding sites for effector molecules (activators and inhibitors). During the AGPase is taken effect by conformational regulation, the main allosteric activator is 3-phosphoglycerate (3-PGA), and the inhibitor is inorganic phosphate (Pi). Through analyzing the AGPase structure and co-crystallization structure of effectors from plants like potatoes and rice, it was revealed that the large subunit proteins provide most of the residues for the effector-binding site (Baris et al., 2009; Maharana et al., 2024). For example, some conservative arginine and lysine residues have positively charged side chains that form ionic and hydrogen bonds with negatively charged effectors (3-PGA and Pi). The small subunit protein contains a highly conserved β-airpin loop (β-T-β), which directly participates in transmitting the allosteric signal, as shown in the β2-TTT-β3 structure (**Figure 2C**). During binding to Pi or absence of effectors, AGPase mainly exists in the T (tense) state form. The subunits protein interface is relatively loose, the catalytic center pocket is relatively closed, the substrate binding affinity is weak, and the activity is low. After combining with 3-PGA, the enzyme shifts towards the R (relaxed) state. The binding of 3-PGA acts like a “molecular glue”, stabilizing the interface between large and small subunits. The stability of the interface is achieved through components like β-hairpin loops, which induce conformational changes in the catalytic center, making its opening and closing more and greatly improving catalytic efficiency (Baris et al., 2009; Maharana et al., 2024).

### 2.3 Spatiotemporal expression patterns of AGPase subunit

**Figure 3:**
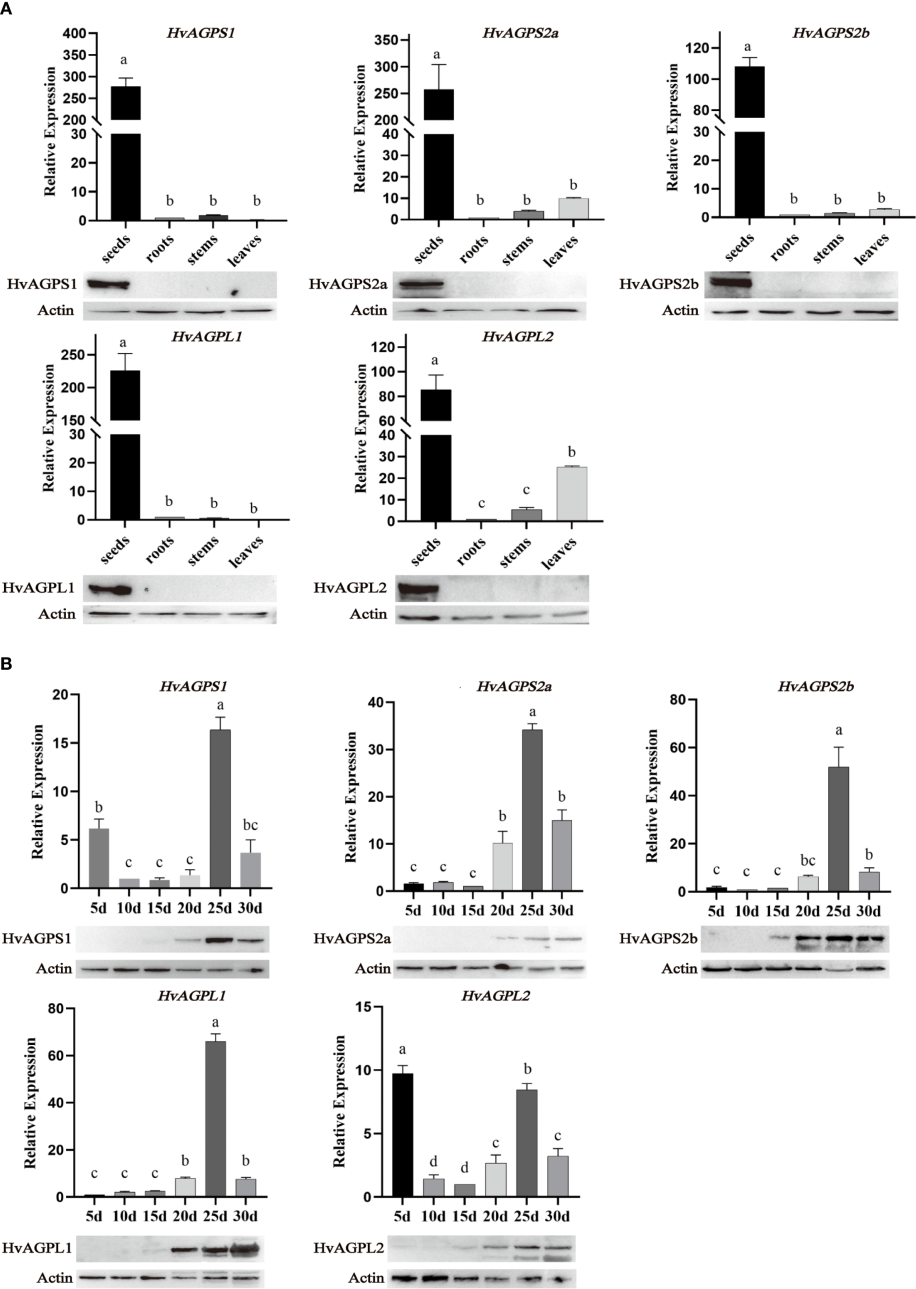
Expression profiles of AGPase subunits. **(A)** The expression of AGPase subunit in different tissues. The expression at the transcriptional level is based on barley actin *HvACT1* as an internal control. The actin expressed at the protein level is plant actin with a dilution of 1:10000, an antibody dilution of 1:2000, and protein loading of 30 μg. The sizes of different subunit proteins in WB: HvAGPS1(55.33kDa); HvAGPS2a(52.03kDa); HvAGPS2b(56.65kDa); HvAGPL1(57.64kDa); HvAGPL2(55.44kDa). **(B)** The expression of AGPase subunit at different stages of grain filling development. Letters (a-d) denote statistically distinct groups (Tukey’s HSD test, p<0.01). Error bars represent ± SD of three biological replicates.

Quantitative real-time PCR analysis revealed distinct tissue-specific expression profiles for all five AGPase subunits (*HvAGPS1, HvAGPS2a, HvAGPS2b, HvAGPL1, HvAGPL2*). Transcript levels in developing grains exceeded those in roots, stems, and leaves by 15- to 32-fold (**Figure 3A**). During grain development, all subunit genes exhibited coordinated transcriptional activation, initiating at 20 DAA, peaking at 25 DAA (with 4.1- to 6.8-fold increases relative to 20 DAA), and subsequently declining (**Figure 3B**). Two subunits displayed unique early expression patterns: *HvAGPS1* transcripts were detectable at 5 DAA (2.3-fold higher than other subunits), followed by *HvAGPL2* at 10 DAA (1.8-fold elevation). Western blot analysis of protein accumulation patterns confirmed and extended these findings (**Figure 3B**). The small subunits protein HvAGPS1 and HvAGPS2b reached maximal abundance at 25 DAA, while HvAGPS2b and HvAGPL2 proteins were first detectable at 15 DAA. In the expression of AGPase subunits in different nutritional tissues (roots, stems, or leaves), our results have demonstrated that *AGPS2a* and *AGPL2* have some expression levels at the transcriptional level, but almost no immunoreactive bands corresponding to AGPase subunits were detected, which may be due to detection sensitivity limitations (**Figure 3A**).

### 2.4 Protein-protein interactions among AGPase subunits

**Figure 4:**
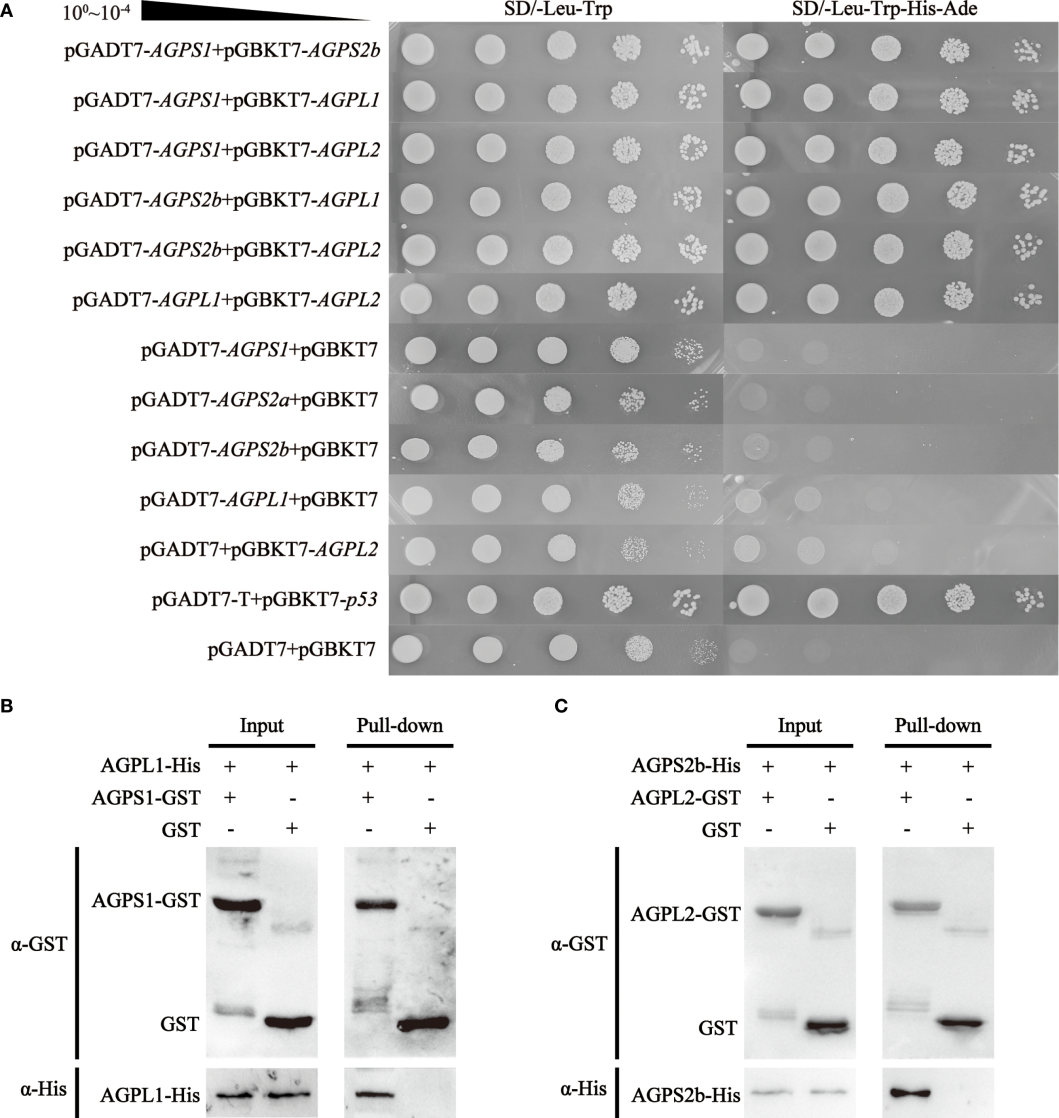
Protein interaction analysis of AGPase subunits. **(A)** Yeast two-hybrid assay demonstrating inter-subunit interactions. Control: SD/-Leu-Trp (double-dropout medium confirming yeast viability). Test: SD/-Leu-Trp-His-Ade (quadruple-dropout medium detecting protein interactions). **(B, C)** GST pull-down validation of heteromeric complexes: **(B)** HvAGPS1-HvAGPL1 complex; **(C)** HvAGPS2b-HvAGPL2 complex. All experiments were replicated three times with <10% variation in band intensity (quantified by ImageJ).

Yeast two-hybrid analysis revealed specific interaction patterns among barley AGPase subunits, demonstrating both heterodimer and homodimer associations. The small subunit protein HvAGPS1 showed selective binding to the large subunit protein HvAGPL1, while HvAGPS2b protein interacted with both HvAGPL1 and HvAGPL2 protein. It was also observed to including SS-SS interactions between HvAGPS1 and HvAGPS2b protein, and LS-LS interactions between HvAGPL1 and HvAGPL2 protein. These interactions were qualitatively confirmed through growth on selective media and α-galactosidase reporter activation in the yeast system (**Figure 4A**). GST pull-down assays provided biochemical validation of key inter-subunit interactions by specific antibodies of AGPase subunits. The results demonstrated that GST-tagged HvAGPS1 specifically pulled down His-tagged HvAGPL1, while His-tagged HvAGPS2b captured GST-tagged HvAGPL2 (**Figures 4B, C**). Control experiments with GST alone showed no detectable binding, confirming the specificity of these interactions. All pull-down experiments were performed in triplicate with consistent results, and bound proteins were detected through immunoblotting with subunit-specific antibodies. The combined results from both yeast two-hybrid and GST pull-down approaches establish that barley AGPase subunits form specific heteromeric complexes, with pairing observed between HvAGPS1-HvAGPL1 and HvAGPS2b-HvAGPL2. These interaction patterns were consistently reproducible across multiple experimental replicates, with less than 10% variation observed between independent trials. Furthermore, although we selected two specific large-small subunit pairs to further validation of their interactions, we do not rule out the potential interactions from combinatorial pairings.

### 2.5 Interactions validation between AGPase subunits

**Figure 5:**
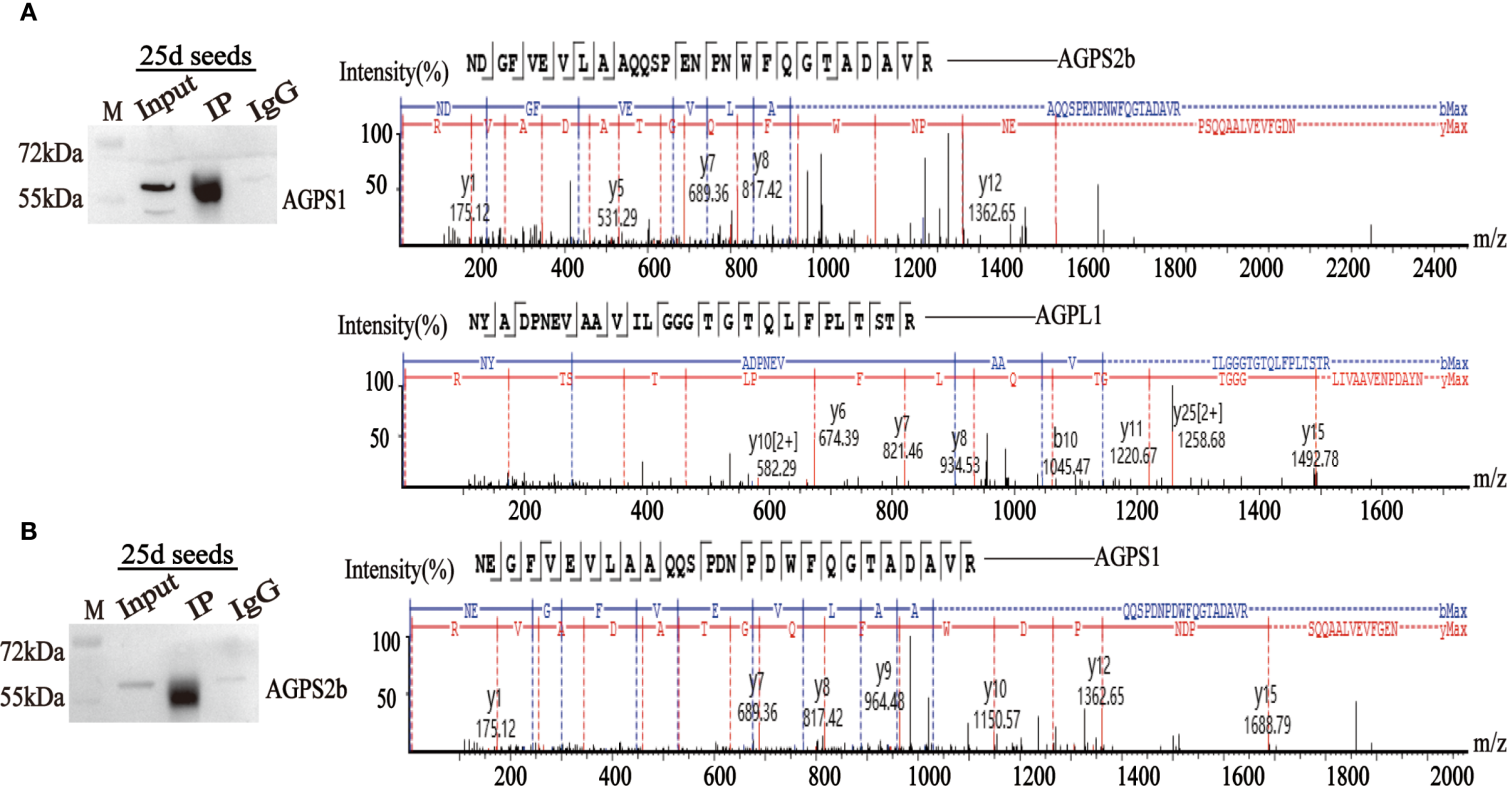
Immunoprecipitation using HvAGPS1 and HvAGPS2b antibodies. **(A)** The mass spectrum of HvAGPS2b and HvAGPL1 protein was screened from the mass spectrometry results of HvAGPS1. **(B)** The mass spectrum of the HvAGPS1 protein was screened from the mass spectrometry results of HvAGPS2b.

Anti-HvAGPS1 antibodies co-precipitated both HvAGPS2b and HvAGPL1, as confirmed by LC-MS/MS analysis with ≥5 unique peptides identified for each interacting protein (FDR < 1%). Reciprocal immunoprecipitation with anti-HvAGPS2b antibodies similarly captured HvAGPS1, demonstrating bidirectional interaction between these small subunits (**Figure 5**). The mass spectrometry data showed significant enrichment of these subunits in immunoprecipitated samples compared to control IgG precipitations (*p* < 0.01, Student’s t-test). The IP-MS results corroborated and GST pull-down assays previous findings from yeast two-hybrid, providing further validation for the following three key interactions: (1) HvAGPS1-HvAGPS2b small subunit heterodimer, (2) HvAGPS1-HvAGPL1 heteromeric complex, and (3) HvAGPS2b-HvAGPL1 interaction. All identified interactions met stringent criteria for identification, including detection in at least two of three biological replicates, a minimum peptide spectrum match value of 20, and absence in negative control samples. The complete mass spectrometry dataset, including peptide counts and statistical confidence metrics, is provided in **Supplementary Table 2**.

### 2.6 *In vitro* enzyme activity determination of different combinations of AGPase subunits

**Figure 6:**
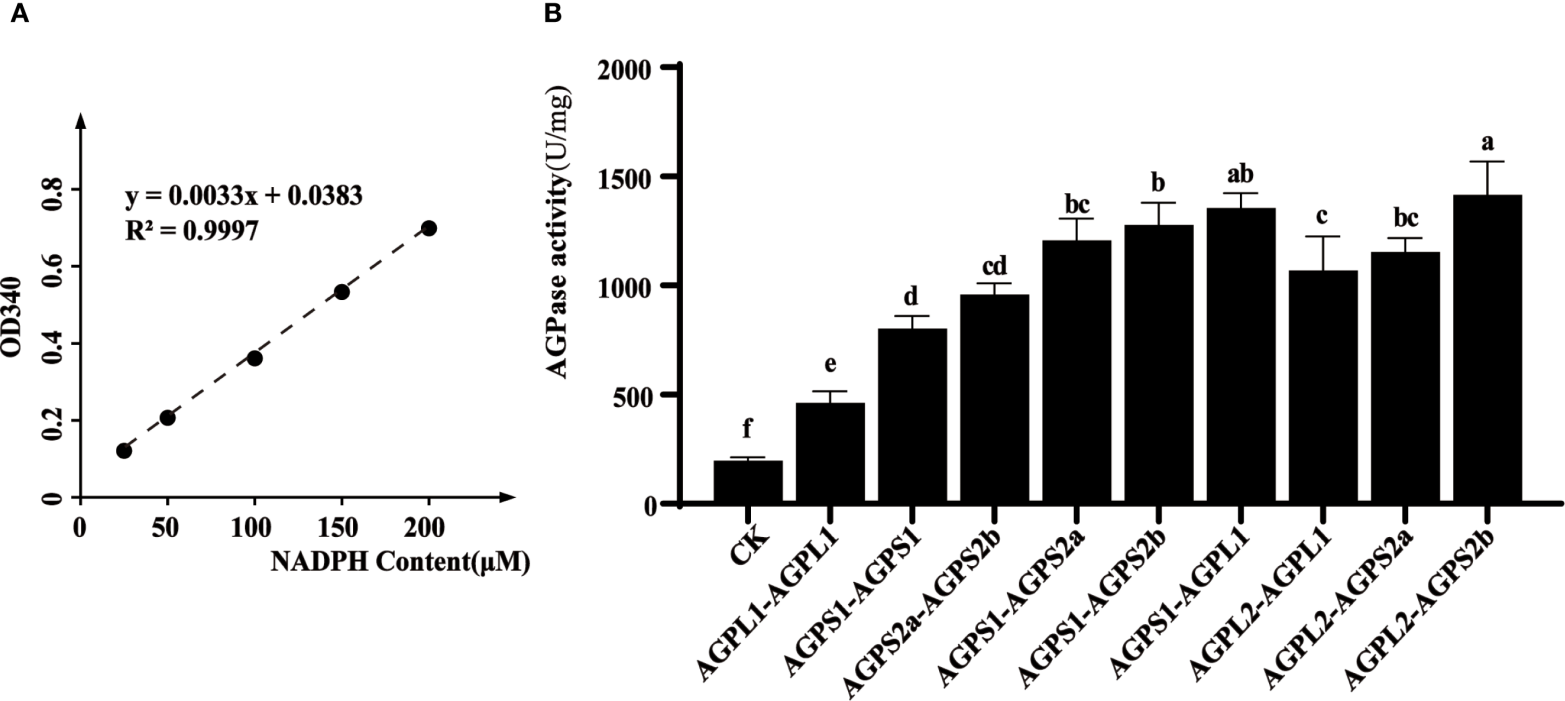
*In vitro* enzymatic activity analysis of AGPase complexes. **(A)** Development of NADPH standard curve. **(B)** Determination of enzyme activity in different combinations. The experimental information on the expression and purification of different subunit proteins is shown in **Supplementary Figure 2**. Letters (a-f) denote statistically distinct groups (Tukey’s HSD test, *p* < 0.01). Error bars represent ± SD of three biological replicates.

*In vitro* enzymatic assays revealed that heterodimeric complexes consistently demonstrated greater activity than homodimeric forms (**Figures 6A, B**). Notably, small subunit homodimers (particularly HvAGPS1) retained measurable catalytic activity, while large subunit homodimers showed minimal function. This observation may reflect the higher structural conservation of small subunits, which contain the essential catalytic domains. Among the various heterodimeric combinations tested, the HvAGPS2b-HvAGPL2 complex displayed the highest specific activity, suggesting this particular subunit pairing may represent the predominant functional form of AGPase in barley. The enhanced activity of heterodimeric complexes compared to homodimers supports the biological importance of proper subunit association for optimal enzyme function.

## 3 Discussion

### 3.1 Spatiotemporal regulation of AGPase gene expression

Our findings demonstrate that barley AGPase subunits exhibit strict tissue-specific expression patterns, with predominant accumulation in developing grains and negligible expression in roots, stems, and leaves. Transcriptional profiling revealed coordinated upregulation of subunit genes during grain filling, peaking at 25 DAA for *HvAGPS1* and *HvAGPS2b* before subsequent decline. This temporal expression pattern differs from related cereals, with maize AGPase activity peaking earlier (~15 DAA) (Na et al., 2018) and wheat showing maximal expression shortly after anthesis (Fahy et al., 2018). Nevertheless, all species demonstrate that elevated AGPase expression correlates strongly with starch accumulation, consistent with wheat studies showing *AGPL1* transcript levels directly proportional to starch synthesis rates (Kumar et al., 2024). At the protein level, barley AGPase subunits first became detectable at 15 DAA, reaching peak abundance by 20 DAA - a pattern generally consistent with transcriptional dynamics. However, we observed several notable exceptions: HvAGPL2 maintained stable protein levels throughout grain filling with minimal fluctuation, while protein accumulation frequently persisted beyond transcriptional downregulation after 30 DAA, likely reflecting the greater stability of mature enzyme complexes. When analyzing incipient the transcriptional expression levels of grains at different developmental stages, it is not difficult to find that early *HvAGPS1* and *HvAGPL2* have significant transcriptional expression levels. It is preliminarily speculated that these early transcripts may play a regulatory or initiatory roles in starch biosynthesis. For example, *ZmAGPL2* is stably expressed throughout grain development in maize, while *AGPS2* is specifically upregulated in the middle and late stages of grain filling, indicating that *AGPL2* may independently participate in early complex pre assembly (Huang et al., 2011). In wheat, *TaAGPS1* is continuously expressed in the early post flowering grains, while *TaAGPL1* expression is lower, suggesting that *AGPS1* may act as a “structural subunit” to initiate complex formation. Therefore, we speculate that as barley belonging to the same family as Poaceae, *HvAGPS1* and *HvAGPL2* have similar functions and roles in early expression. At the same time, when detecting AGPase activity *in vivo*, it was found that there was partial AGPase activity during the early stages of endosperm development, but a considerable lack of protein levels was observed at the same developmental stage (**Figures 1A**, **4B**). This may be attributed to the partial expression of *HvAGPS1* and *HvAGPL2*, as well as the trace expression of other subunits, which provides considerable AGPase activity during early grain development. This partial AGPase activity can meet the requirements of early starch synthesis and prevent excessive accumulation of monosaccharides in the grain (Huang et al., 2011). The observed spatiotemporal expression patterns suggest an elaborate regulatory network coordinating AGPase production with starch biosynthesis demands during grain development. The persistence of AGPase proteins beyond their transcriptional peak may represent an adaptive mechanism to maintain starch production during late grain filling stages.

The multi-band phenomenon observed for AGPS2b and AGPL2 in Western Blot (WB) experiments, which suggests the existence of multiple proteins forms. The appearance of multiple bands in AGPS2b and AGPL2 samples indicates the presence of a group of mature and immature proteins containing transport peptides, implying that these isoforms are localized to plastids. In previous studies on AGPase in cereal endosperms, unlike many other plant tissues, the majority of the AGPase activity was found to reside in the cereal endosperm exists in the cytoplasm, while a small portion located within plastids. In maize endosperm, the primary AGPase enzyme activity is present in the cytoplasm, with SH2 (LSU) and BT2 (SSU) being the main cytoplasmic AGPase subunits. However, some subunits are also transported to the plastid through plastid transport peptides. The cytoplasmic LSU binds to the SSU precursor protein carrying a transport peptide to form a heterodimeric complex (LSU-SSU precursor), which is recognized by proteins on the plasma membrane through the transport peptide and transported into the plastid. After this complex entering the plastid, the transport peptide is cleaved and assembled with the LSU inside the plastid to form the final active heterotetramer (LSU_2_SSU_2_). Therefore, this portion of AGPase subunits is ultimately localized in the plastid stroma (Yu et al., 2023b; Huang et al., 2011).

### 3.2 Cross-reactivity between AGPase subunit antibodies and other subunit proteins

During preparing exogenous antibodies against AGPase subunits, cross-reactivity between antibodies and other subtype subtypes is a common phenomenon, particularly in graminaceous plants. This cross-reactivity is mainly attributed to high sequence homology among subunits and the overlap of conserved Motifs. For example, the homologous alignment rate has 82% between ZmAGPL2 and ZmAGPL1 in N-terminal 1–150 amino acid, and the antibody epitope is often located in this region. Moreover, all AGPase subunits contain 10 evolutionarily conserved motifs, and their relative positions and lengths are strictly conserved. If the antibody targets these regions, it is easy to cross-react with different subtypes. Although the phenomenon of antibody cross-reactivity between AGPase subunit proteins is difficult to handle, it also indirectly reveals the evolutionary conservation and functional redundancy of AGPase subunits. For example, the cross-reactivity of ZmAGPL2 antibody with the ancestral gene *ZmLSU3* supports that the AGPase subunit of Poaceae originated from a common ancestor. Similarly, through the phenomenon of OsAGPS2b antibody misidentifying leaf *OsAGPS1* indicates functional redundancy between these two subunits in photosynthetic carbon allocation.

### 3.3 Subunit interaction dynamics of barley AGPase

Our comprehensive *in vitro* characterization of AGPase subunit association provides significant insights into the molecular architecture of this critical enzyme complex in barley. The GST pull-down assays validated the yeast two-hybrid results, confirming a stable interaction between HvAGPS2b and HvAGPL2, consistent with observations in other cereals where similar subunit combinations form functional heterodimers (Gann et al., 2020). However, our IP-MS analysis revealed a more complex interaction landscape than previously recognized, identifying both heteromeric and homomeric subunit associations. The detection of HvAGPS1-HvAGPS2b interactions suggests potential small subunit oligomerization, while the variable recovery of HvAGPL2 in IP-MS experiments despite positive yeast two-hybrid results points to context-dependent regulation of subunit hetero-oligomerization. This discrepancy may reflect several biological realities: the relatively low expression of *HvAGPL2* transcripts compared to other subunits, potential post-translational modifications that modulate interaction stability (Wei et al., 2017), or the formation of transient complexes that are challenging to capture under experimental conditions. The core reason is that the transient complexes formed by HvAGPL2 protein and other subunits are difficult to stably capture in IP (Berggård et al., 2007). Besides, the identification of HvAGPL1 in HvAGPS2b immunoprecipitates, despite its absence from yeast two-hybrid interactions, further underscores the complexity of AGPase assembly and suggests that native cellular environments may facilitate interactions not observed in heterologous systems. These findings collectively indicate that barley AGPase likely exists as a dynamic ensemble of complexes whose composition may vary according to developmental stage, subcellular localization, and metabolic demands. The demonstration of multiple interaction patterns challenges the conventional view of AGPase as a simple heterotetramer and suggests a more sophisticated regulatory mechanism governing its assembly and function in starch biosynthesis.

### 3.4 Functional characterization of AGPase subunit combinations

Our investigation of AGPase enzymatic properties during grain development revealed a distinct bell-shaped activity profile that closely paralleled starch accumulation patterns. *In vitro* biochemical characterization demonstrated significant variation in catalytic efficiency among different subunit combinations, with heterodimeric complexes consistently outperforming homodimeric forms. Notably, the HvAGPS2b-HvAGPL2 heterodimer exhibited the highest specific activity, suggesting this pairing represents the predominant functional configuration in barley, as previously observed in other cereals (Seferoglu et al., 2016). Comparative analysis revealed that small subunit homodimers retained measurable activity while large subunit homodimers showed minimal catalytic function, indicating their structural instability in isolation. These functional differences correlate with evolutionary patterns observed at the molecular level. Small subunits display remarkable sequence conservation across species, reflecting stringent structural constraints required for maintaining catalytic competence (Maharana et al., 2024). In contrast, large subunits exhibit greater sequence variability, consistent with their primary role in regulatory adaptation rather than direct catalysis. Our findings support the model where proper subunit stoichiometry and interaction geometry are critical for optimal enzyme function - imbalances disrupt the essential quaternary structure and impair activity (Hsu et al., 2022). The C-terminal domains of small subunits appear particularly crucial for complex assembly, as demonstrated by studies showing that truncation of these regions in rice AGPS compromises enzyme integrity (Maharana et al., 2024; Ohdan et al., 2005). These structural-functional relationships explain why natural selection maintains specific pairing preferences despite the combinatorial possibilities offered by multiple subunit isoforms. Among them, natural selection maintains the preferred pairing of AGPase subunits. In the process of domestication or breeding, the preference for subunit combinations is inevitably influenced. For example, the critical combinationship of AGPL2 and AGPS2b was disrupted in the maize *Sh2* mutant, leading to an imbalance dysregulation of carbon source allocation in endosperm, resulting in a 30% decrease in seed germination rate and requiring artificial seedling cultivation (Dong et al., 2019). Conversely, Edited promoter of *OsWx* gene to adapt to rice cooking preferences in rice, resulting in an increased expression of *OsAGPL2* in the combination of *OsAGPL2* and *OsAGPS2b*, ultimately producing economically valuable low-amylose varieties (Maharana et al., 2024). Consequently, it can be seen that changes in AGPase subunit combinations during domestication or breeding can pierce limitations that natural selection cannot achieve.

## 4 Conclusion

This study provides a comprehensive understanding of the molecular and functional characteristics of AGPase in barley, revealing its critical role in starch biosynthesis. Phylogenetic analysis confirmed the close evolutionary relationship between barley and wheat AGPase subunits, with high sequence homology and conserved structural motifs, suggesting shared ancestry and functional conservation. Expression profiling demonstrated that AGPase subunits are predominantly active during grain filling (20–30 DAA), with protein and transcript levels peaking in synchrony, underscoring their importance in mid-to-late grain development. *In vitro* enzymatic assays revealed that heterodimeric complexes, particularly the HvAGPS2b-HvAGPL2 combination, exhibit significantly higher activity than homodimers, surmising this pairing as the most catalytically efficient configuration in barley. Protein interaction analyses, including yeast two-hybrid, GST pull-down, and immunoprecipitation-mass spectrometry, validated both heteromeric (HvAGPS1-HvAGPL1, HvAGPS2b-HvAGPL2) and homomeric (SS-SS, LS-LS) interactions, highlighting the dynamic assembly of AGPase complexes. This also implies that AGPase likely forms different complexes to control enzyme activity and thereby regulate starch synthesis. These findings collectively demonstrate that barley AGPase operates through a tightly regulated, evolutionarily conserved interaction network, where specific subunit combinations optimize enzymatic efficiency and drive starch accumulation during grain filling. Based on these characteristics, molecular markers could be designed to efficiently screen barley germplasm carrying highly active complexes (as HvAGPS2b-HvAGPL2). Concurrently, when cultivating barley varieties with cooking preferences, targeted editing the promoter of *HvAGPL2* gene may change the expression ratio of *HvAGPL2* and *HvAGPS2b*, and offer a viable strategy for low-starch or high-starch cultivars.

## 5 Material and methods

### 5.1 Plant materials and growth conditions

The study utilized barley (*Hordeum vulgare* L.) cultivar ‘Damai Kangqing 9’ grown under standard field conditions at the Barley Research Base of Sichuan Agricultural University during the 2022–2023 growing season. For temporal expression analysis, developing grains were systematically collected at six key developmental stages: 5, 10, 15, 20, 25, and 30 days after anthesis (DAA), with anthesis date determined by visual examination of spike development (Yu et al., 2023a; Huang et al., 2011). Concurrently at 25 DAA, vegetative tissues including roots (primary and secondary roots from 0–20 cm soil depth), stems (second internode from apex), and leaves (fully expanded flag leaves) were harvested for spatial expression profiling. All samples were immediately flash-frozen in liquid nitrogen and stored at -80°C until analysis. Three biological replicates were collected for each time point and tissue type, with each replicate consisting of pooled material from 10 randomly selected plants to account for biological variability. Field management followed standard agronomic practices for barley production in the region, including optimal fertilization (300 kg/ha NPK 15:15:15), controlled irrigation (maintaining 70-80% field capacity), and integrated pest management with minimal chemical intervention.

### 5.2 Bioinformatics analysis

Phylogenetic reconstruction of AGPase subunit evolution was performed using MEGA-X software (version 11.0.13) with the following analytical parameters: (1) amino acid sequences of both large and small AGPase subunits were retrieved from four Poaceae species - barley, wheat (*Triticum aestivum* L.), rice (*Oryza sativa* L.), and maize (*Zea mays* L.); (2) multiple sequence alignment was conducted using the MUSCLE algorithm with default parameters; (3) phylogenetic trees were constructed using the maximum likelihood method with 1000 bootstrap replicates to assess node support (Xi et al., 2024). For structural characterization, protein sequences of all barley AGPase subunits were analyzed using ESPript 3.0 to generate multiple sequence alignments with secondary structure annotations. Furthermore, conserved motifs were identified through the MEME online suite (version 5.5.2) with the following search parameters: (i) maximum number of motifs set to 10, (ii) minimum motif width of 6 residues, (iii) maximum motif width of 50 residues, and (iv) E-value threshold of 1×10^-10^. All sequence data were obtained from the NCBI protein database with rigorous verification of annotation accuracy before analysis.

### 5.3 Transcript-level expression analysis *via* qRT-PCR

Gene-specific primers for AGPase subunits were designed using Primer Premier 5.0 software with the following parameters: amplicon length 80–150 bp, melting temperature 58-62 °C, and GC content 40-60% (Liu et al., 2023). Total RNA was extracted from various tissues (roots, stems, leaves) and developing grains (5–30 DAA) using the RNAprep Pure Plant Plus Kit (Cwbio), followed by DNase I treatment to eliminate genomic DNA contamination. RNA integrity was verified by 1.2% agarose gel electrophoresis and quantified using a NanoDrop spectrophotometer (OD260/280 ratio >1.9). First-strand cDNA synthesis was performed with 1 μg total RNA using the HiScript III RT SuperMix (Cwbio) according to the manufacturer’s protocol. Quantitative real-time PCR was conducted on a CFX96 Touch system (Bio-Rad) with the following cycling conditions: 95 °C for 30 sec, followed by 40 cycles of 95 °C for 10 sec and 60 °C for 30 sec, using SYBR Green Master Mix (GeneStar). Each reaction (20 μL) contained 10 ng cDNA template, 0.5 μM of each primer, and 1× SYBR Green Master Mix. The barley β-actin gene served as the internal reference for normalization. Three technical replicates were performed for each biological sample (n=3), with relative expression levels calculated using the 2-ΔΔCt method. Melting curve analysis (65-95 °C) confirmed amplification specificity, and primer efficiencies (90-110%) were validated through standard curves. Negative controls (no-template and no-RT) were included in each run to ensure the absence of contamination.

### 5.4 Western blot analysis

Protein samples were separated by 10% SDS-PAGE electrophoresis (100 V, 90 min) and subsequently transferred to PVDF membranes (0.45 μm pore size) using a wet transfer apparatus (Bio-Rad) at 100 V for 1 hour in transfer buffer (25 mM Tris, 192 mM glycine, 20% methanol). Following transfer, membranes were briefly rinsed with TBST buffer (20 mM Tris-HCl, pH 7.5, 150 mM NaCl, 0.1% Tween-20) and blocked with 5% (w/v) non-fat dry milk in TBST for 1 hour at room temperature with gentle agitation (Shoaib et al., 2023). After three 5-minute washes with TBST, membranes were incubated with primary antibodies (rabbit polyclonal anti-AGPase subunits, 1:2000 dilution in blocking buffer) for 2 hours at room temperature. Following primary antibody incubation, membranes were washed three times (10 min each) with TBST and then probed with HRP-conjugated goat anti-rabbit secondary antibody (1:5000 dilution in blocking buffer) for 1 hour. The primary antibody is prepared using the previously prepared antibody, and its preparation is based on the article in Xi (Xi et al., 2024). After three final TBST washes (10 min each), protein bands were visualized using the Sheng’er Biochemical Luminescence Kit (SB-WB004) according to the manufacturer’s instructions, with chemiluminescent signals captured by a CCD imaging system (Tanon 5200). Image analysis was performed using ImageJ software (NIH) with normalization to actin (mouse monoclonal anti-actin, 1:10000 dilution) as a loading control.

### 5.5 Co-immunoprecipitation mass spectrometry analysis

Protein extracts from developing barley grains (25 DAA) were pre-cleared by incubation with rabbit IgG-conjugated Protein A/G magnetic beads (Thermo Fisher Scientific) in lysis buffer (50 mM Tris-HCl pH 7.5, 150 mM NaCl, 1% Triton X-100, 1× protease inhibitor cocktail) at 4 °C for 1 hour with end-over-end rotation (Zhou et al., 2024). For immunoprecipitation, 20 μL of antibody-conjugated beads (anti-HvAGPS1 protein or anti-HvAGPS2b protein) were added to 500 μL pre-cleared lysate (1 mg/mL total protein) and incubated overnight at 4 °C with constant agitation. Beads were subsequently collected by magnetic separation and washed three times with 0.5 mL ice-cold lysis buffer under mild denaturing conditions (0.1% SDS). Bound proteins were eluted with 50 μL of 0.1 M glycine-HCl (pH 2.5) and immediately neutralized with 1 M Tris-HCl (pH 8.0). Immunoprecipitation efficiency was validated by Western blotting before MS analysis. For LC-MS/MS, eluted proteins were precipitated using trichloroacetic acid/acetone, reduced with 10 mM DTT, alkylated with 55 mM iodoacetamide, and digested with trypsin (1:50 w/w) overnight at 37 °C. Peptide mixtures were analyzed by nanoLC-MS/MS (Q Exactive HF-X, Thermo Scientific) with a 120-min gradient (5-35% acetonitrile in 0.1% formic acid) at a flow rate of 300 nL/min. MS data were acquired in data-dependent acquisition mode with the following parameters: MS1 resolution 60,000, MS2 resolution 15,000, top 20 precursor ions selected for fragmentation. Protein identification and interaction partner analysis were performed using MaxQuant (v2.0.3.0) against the UniProt *Hordeum vulgare* database (release 2023_01), with false discovery rate (FDR) set to 1% at both peptide and protein levels.

### 5.6 Yeast two-hybrid assay

The coding sequences of AGPase subunits (*HvAGPS1*, *HvAGPS2b*, *HvAGPL1*, *HvAGPL2*) were cloned into either the pGBKT7 bait vector (DNA-binding domain) or pGADT7 prey vector (activation domain) using standard restriction enzyme digestion and ligation methods (Zhang et al., 2024). Yeast strain AH109 was co-transformed with bait-prey plasmid combinations and selected on SD/-Leu/-Trp medium to confirm successful co-transformation. Protein-protein interactions were assessed by plating transformants on stringent SD/-Leu/-Trp/-His/-Ade medium supplemented with X-α-Gal, with positive interactions indicated by colony growth and blue coloration after 5 days of incubation at 30°C. Appropriate controls were included in all experiments: pGBKT7-53 + pGADT7-T served as a positive control, pGBKT7-Lam + pGADT7-T as a negative control, and all bait constructs were tested for autoactivation by transformation without prey vectors.

### 5.7 GST pull-down assay

Protein-protein interactions were validated using GST pull-down assays with purified recombinant proteins. His-tagged AGPase subunits (10 μg) were incubated with glutathione-sepharose beads pre-bound to GST-fusion proteins (20 μg) in binding buffer (50 mM Tris-HCl pH 7.5, 150 mM NaCl, 1% Triton X-100) for 12 hours at 4°C with end-over-end rotation. Following incubation, beads were pelleted by centrifugation (500*g, 5 min, 4°C) and washed three times with ice-cold wash buffer (0.1% SDS binding buffer). Bound protein complexes were eluted by boiling in 2× SDS loading buffer for 5 minutes and resolved by 12% SDS-PAGE. Proteins were transferred to PVDF membranes using a semi-dry transfer apparatus (25 V, 30 min). Afterwards, subjected to immunoblot analysis with mouse anti-GST (1:5,000) and rabbit anti-His (1:3,000) primary antibodies, followed by HRP-conjugated secondary antibodies (1:10,000). Signal detection was performed using enhanced chemiluminescence substrate with exposure times ranging from 30 sec to 5 min (Sambrook and Russell, 2006; Nixon et al., 2002). Control experiments included: (1) GST-only beads with His-tagged proteins to assess nonspecific binding, and (2) GST-fusion proteins with non-recombinant E. coli lysate to confirm specificity. All pull-down experiments were performed in triplicate with consistent results (CV < 15% between replicates). Band intensities were quantified using ImageJ software (NIH) with background subtraction.

### 5.8 AGPase activity and starch content

Following the method described by Nishi (Nishi et al., 2001), 0.05g of shelled seeds were ground using a pestle and diluted in 200 μL of equilibration buffer (pH=7.4, 50 mM HEPES, 5 mM MgCl_2_, and 0.5 mM EDTA) and centrifuged for clarification. The final volume is used to calculate the activity of the Unit endosperm. Add 2 mL of HQ-A buffer (pH=6.8, 50 mM potassium phosphate, 5 mM MgCl_2_, and 0.5 mM EDTA) and centrifuge at 15000*g for 5 minutes at 4°C. Take 1 mL of supernatant and precipitate it with 45% ammonium sulfate. Resuspend the precipitate in 200 μL of HQ-A buffer. After resuspension, the sample was heat-treated at 60°C for 7 minutes, cooled, and centrifuged at 15000*g for 5 minutes. The activity assay was conducted at 37°C (incubation time of 6 minutes), and the control reaction system included all substrate mixtures except PPi. After the reaction was terminated, the NADPH content was measured by adding 500 μL of colorimetric mixture (pH=7.4100 mM MOPS HCl, 0.1 mg/mL BSA, 7 mM MgCl_2_ and 0.6 mM NADP, 1 Unit of glucose-6-phosphate dehydrogenase, 1 Unit of phosphoglucose mutase). After centrifugation and clarification for 5 minutes, the absorbance of the reaction solution was measured at a wavelength of 340 nm. The generated Glc-1P content is determined by a standard curve, which is plotted using a freshly prepared Glc-1-P complete reaction system without enzymes. Specific activity is defined as the number of Units per milligram of protein, where 1 Unit refers to the amount of enzyme that catalyzes the conversion of 1 μ mol substrate per minute. *In vitro* experiments, a total of 30uL of purified elution mixture was added to the equilibrium buffer in a 1:1 ratio of subunits within each combination, calculated by ImageJ. The remaining steps were performed *in vivo* experiments. The starch content was determined by using the total starch measurement kit from Beijing Solaibao Technology Co., Ltd.

## Data Availability

The original contributions presented in the study are included in the article/**Supplementary Material**. Further inquiries can be directed to the corresponding author.
