# Supplementary figures and images for: Expression and interaction of AGPase subunits reveal functional enzyme complexes in barley
Source: Front Plant Sci. 2025 Oct 16;16:1671162. doi: 10.3389/fpls.2025.1671162 (PMC12571849; doi:10.3389/fpls.2025.1671162)

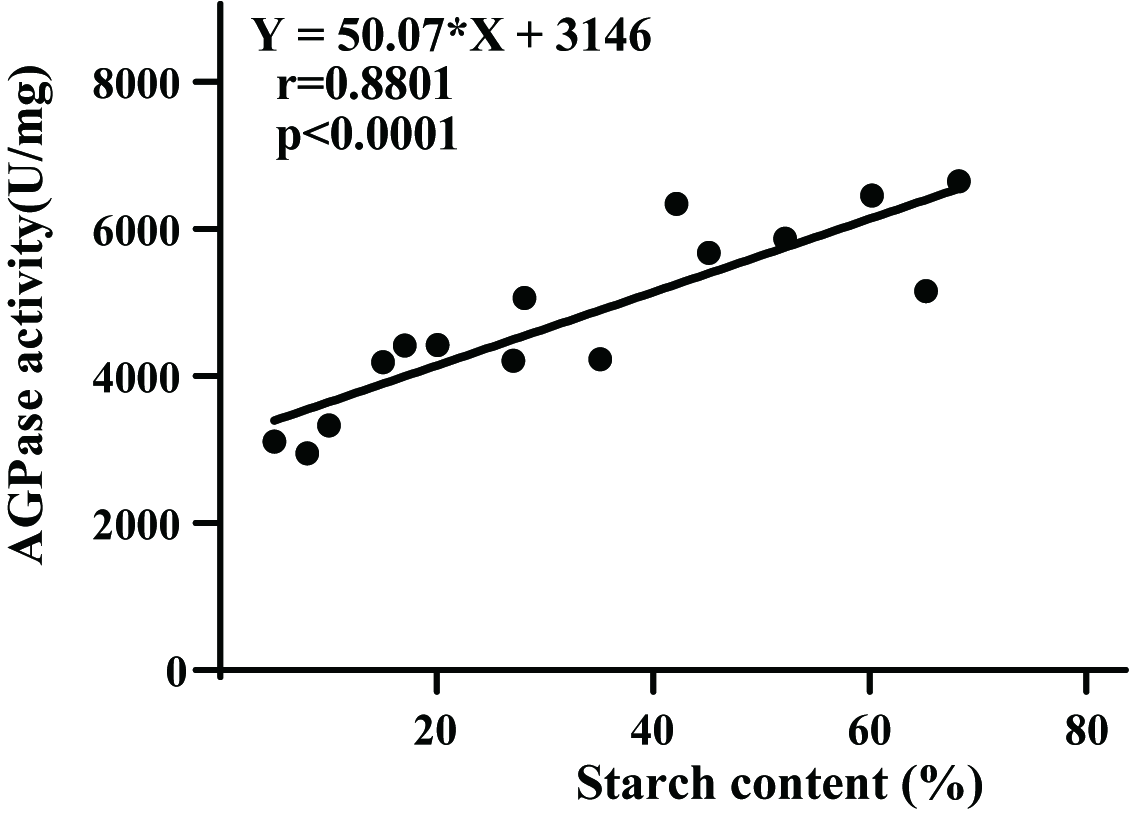

Supplement: Supplementary file 1 [file Image1.tif]

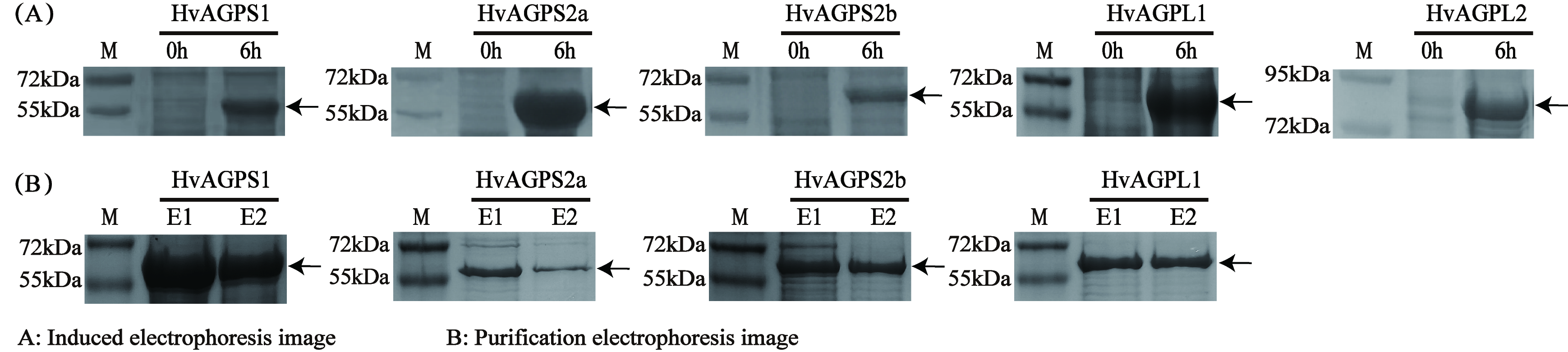

Supplement: Supplementary file 2 [file Image2.tif]

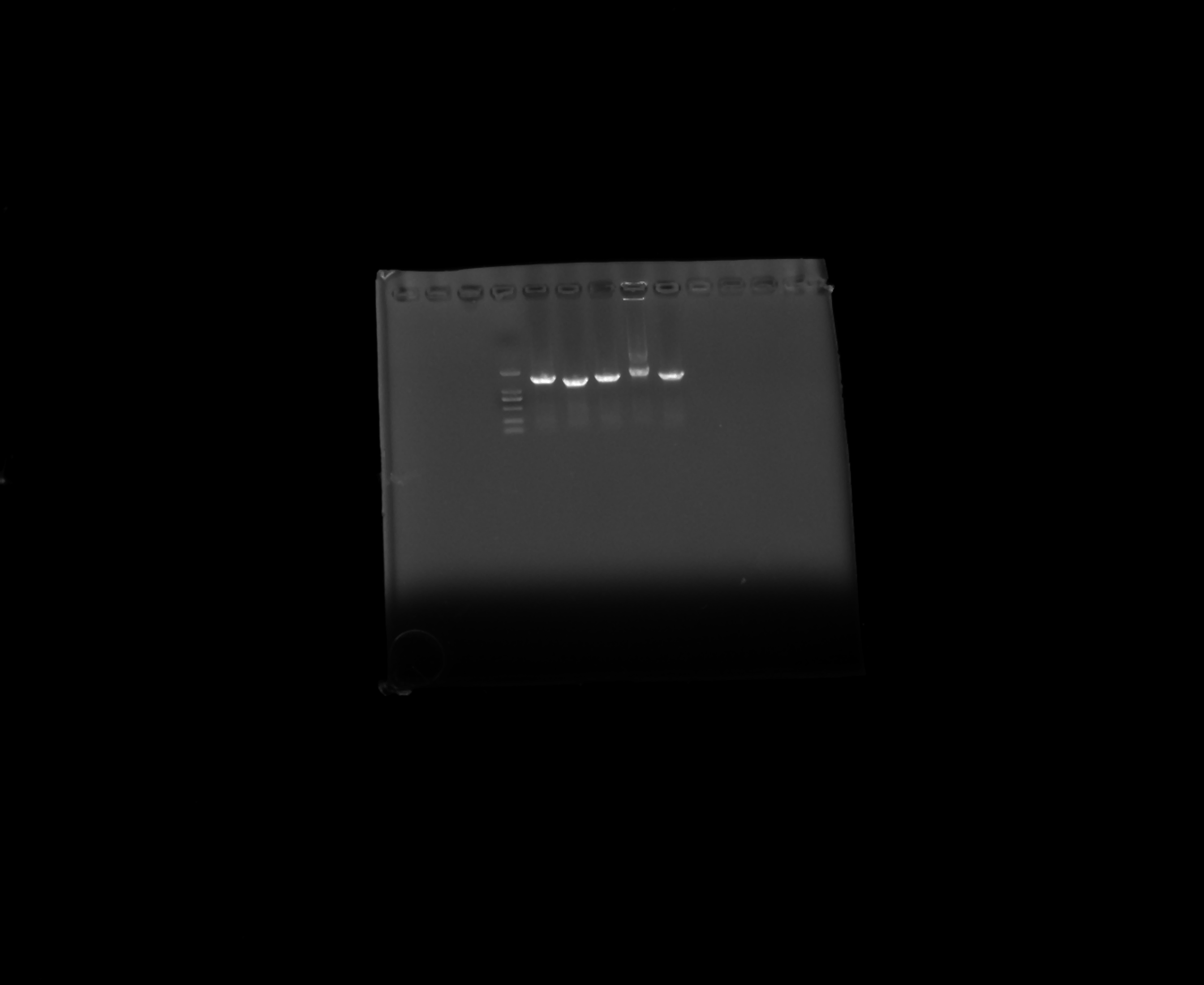

Supplement: Supplementary file 6 [file DataSheet1.zip › 1/Clone/20250208-123756[Gel].tif]

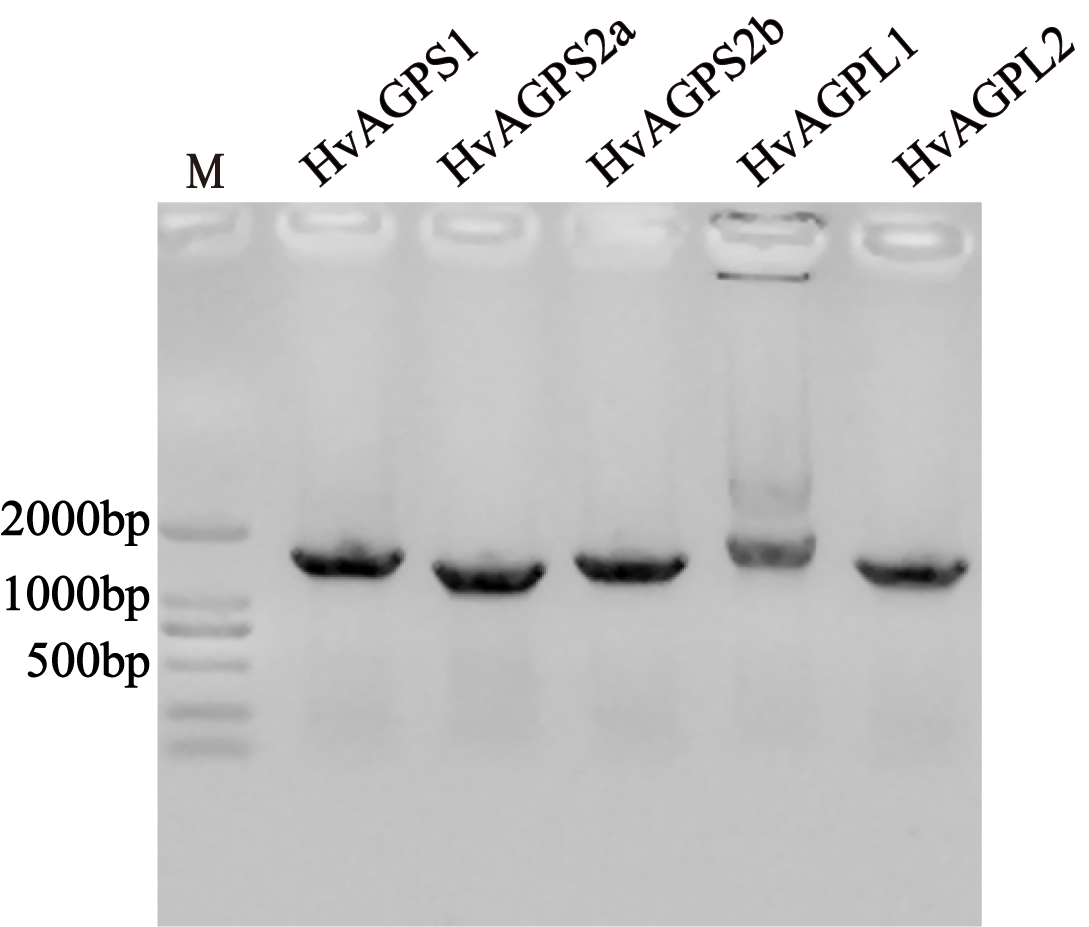

Supplement: Supplementary file 6 [file DataSheet1.zip › 1/Clone/克隆1.tif]

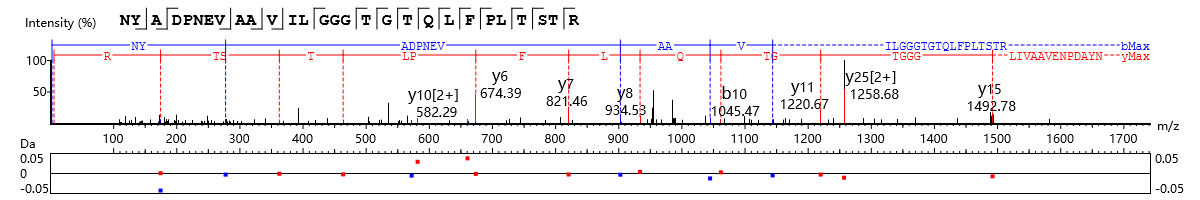

Supplement: Supplementary file 6 [file DataSheet1.zip › 1/CoIP-MS/AGPS1 to AGPL1.png]

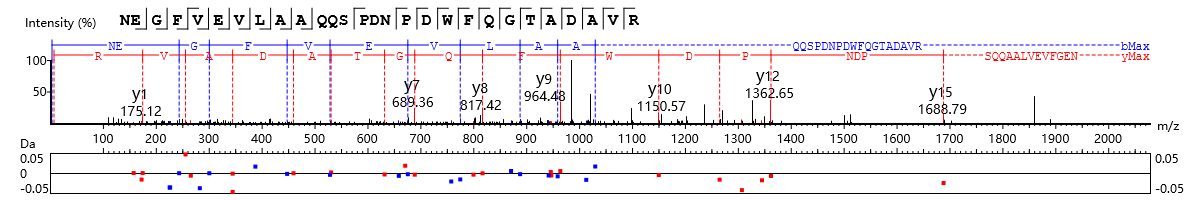

Supplement: Supplementary file 6 [file DataSheet1.zip › 1/CoIP-MS/AGPS1 to AGPS2b.tif]

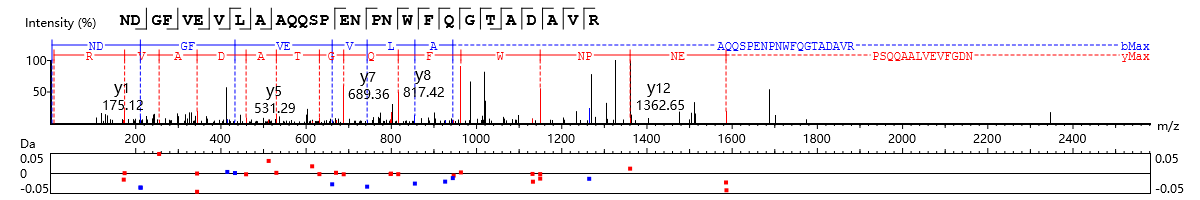

Supplement: Supplementary file 6 [file DataSheet1.zip › 1/CoIP-MS/AGPS2b to AGPS1.png]

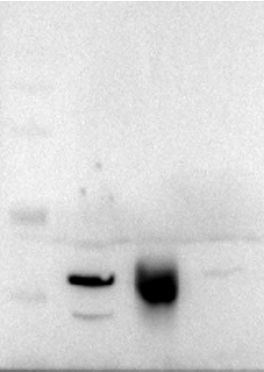

Supplement: Supplementary file 6 [file DataSheet1.zip › 1/CoIP-MS/IP-WB/AGPS1.png]

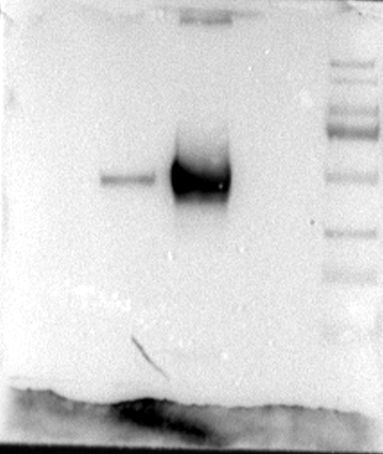

Supplement: Supplementary file 6 [file DataSheet1.zip › 1/CoIP-MS/IP-WB/AGPS2a.tif]

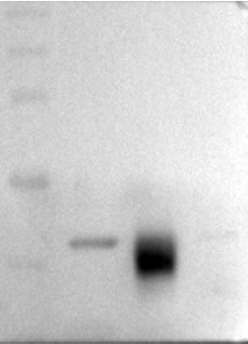

Supplement: Supplementary file 6 [file DataSheet1.zip › 1/CoIP-MS/IP-WB/AGPS2b.png]

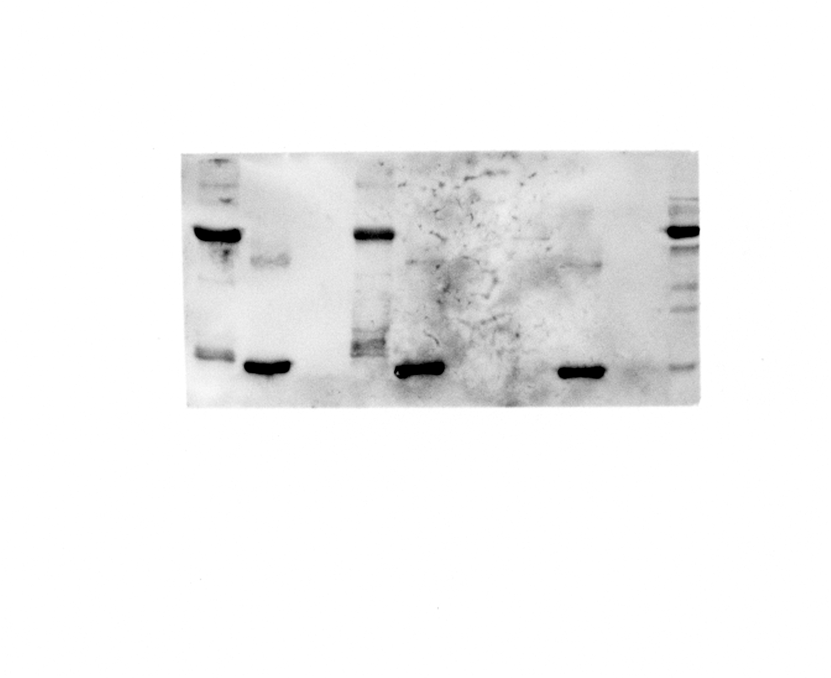

Supplement: Supplementary file 6 [file DataSheet1.zip › 1/GST-Pull down/S1-L1.tif]

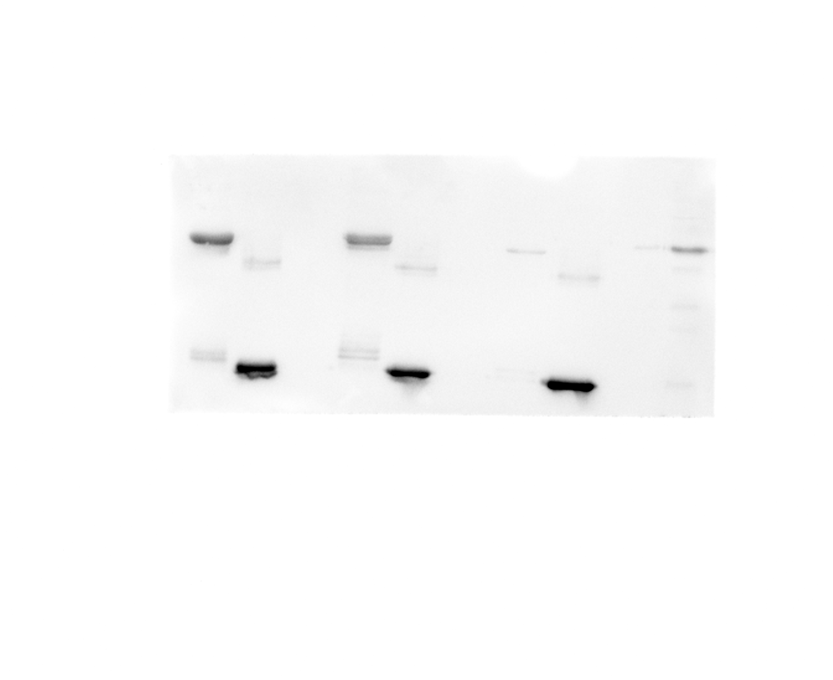

Supplement: Supplementary file 6 [file DataSheet1.zip › 1/GST-Pull down/S2b-L2.tif]

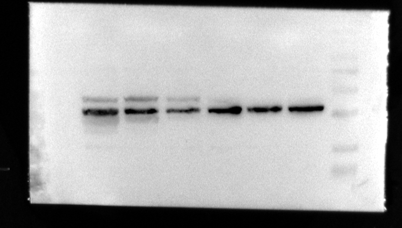

Supplement: Supplementary file 6 [file DataSheet1.zip › 1/Spatiotemporal Expression/Protein expression/different stages of grain filling development/AGPL1-actin.tif]

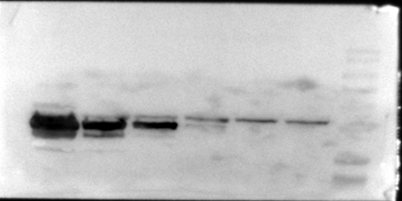

Supplement: Supplementary file 6 [file DataSheet1.zip › 1/Spatiotemporal Expression/Protein expression/different stages of grain filling development/AGPL1.tif]

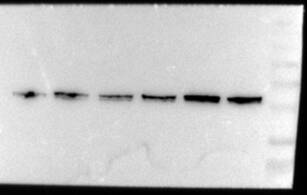

Supplement: Supplementary file 6 [file DataSheet1.zip › 1/Spatiotemporal Expression/Protein expression/different stages of grain filling development/AGPL2-actin.tif.jpg]

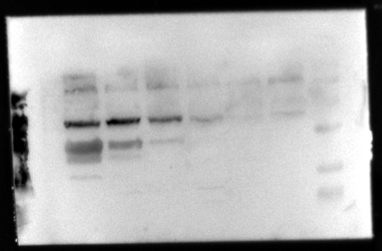

Supplement: Supplementary file 6 [file DataSheet1.zip › 1/Spatiotemporal Expression/Protein expression/different stages of grain filling development/AGPL2.tif]

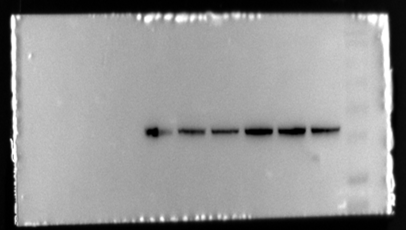

Supplement: Supplementary file 6 [file DataSheet1.zip › 1/Spatiotemporal Expression/Protein expression/different stages of grain filling development/AGPS1-actin.tif]

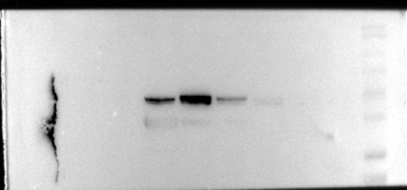

Supplement: Supplementary file 6 [file DataSheet1.zip › 1/Spatiotemporal Expression/Protein expression/different stages of grain filling development/AGPS1.tif]

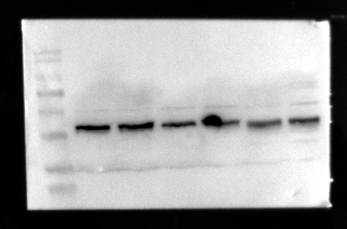

Supplement: Supplementary file 6 [file DataSheet1.zip › 1/Spatiotemporal Expression/Protein expression/different stages of grain filling development/AGPS2a-actin.tif]

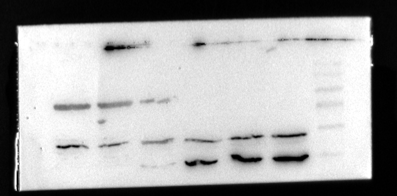

Supplement: Supplementary file 6 [file DataSheet1.zip › 1/Spatiotemporal Expression/Protein expression/different stages of grain filling development/AGPS2a.tif]

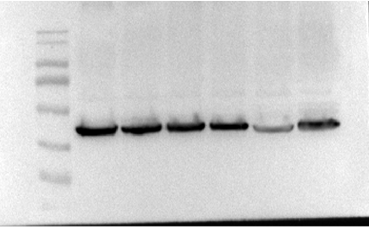

Supplement: Supplementary file 6 [file DataSheet1.zip › 1/Spatiotemporal Expression/Protein expression/different stages of grain filling development/AGPS2b-actin.png]

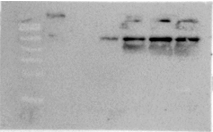

Supplement: Supplementary file 6 [file DataSheet1.zip › 1/Spatiotemporal Expression/Protein expression/different stages of grain filling development/AGPS2b.png]

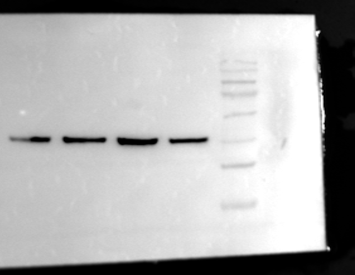

Supplement: Supplementary file 6 [file DataSheet1.zip › 1/Spatiotemporal Expression/Protein expression/different tissues/AGPL1-actin.tif]

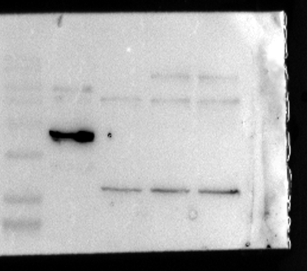

Supplement: Supplementary file 6 [file DataSheet1.zip › 1/Spatiotemporal Expression/Protein expression/different tissues/AGPL1.tif]

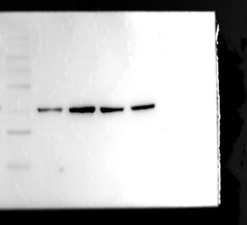

Supplement: Supplementary file 6 [file DataSheet1.zip › 1/Spatiotemporal Expression/Protein expression/different tissues/AGPL2-actin.tif]

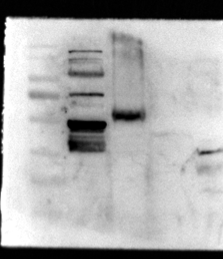

Supplement: Supplementary file 6 [file DataSheet1.zip › 1/Spatiotemporal Expression/Protein expression/different tissues/AGPL2.tif]

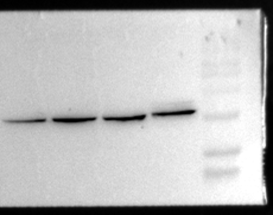

Supplement: Supplementary file 6 [file DataSheet1.zip › 1/Spatiotemporal Expression/Protein expression/different tissues/AGPS1-actin.tif]

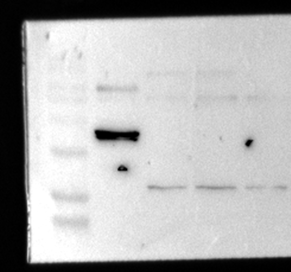

Supplement: Supplementary file 6 [file DataSheet1.zip › 1/Spatiotemporal Expression/Protein expression/different tissues/AGPS1.tif]

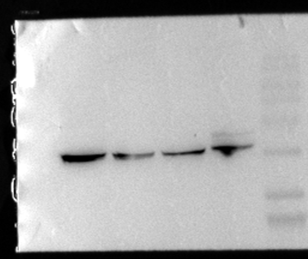

Supplement: Supplementary file 6 [file DataSheet1.zip › 1/Spatiotemporal Expression/Protein expression/different tissues/AGPS2A-actin.tif]

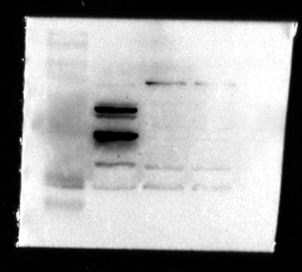

Supplement: Supplementary file 6 [file DataSheet1.zip › 1/Spatiotemporal Expression/Protein expression/different tissues/AGPS2A.tif]

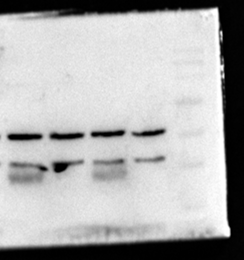

Supplement: Supplementary file 6 [file DataSheet1.zip › 1/Spatiotemporal Expression/Protein expression/different tissues/AGPS2b-actin.tif]

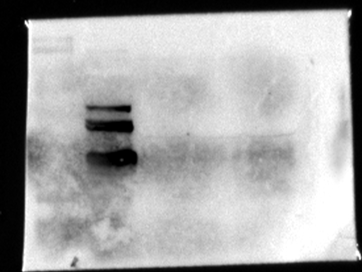

Supplement: Supplementary file 6 [file DataSheet1.zip › 1/Spatiotemporal Expression/Protein expression/different tissues/AGPS2b.tif]
