# Supplementary material for: Expression and interaction of AGPase subunits reveal functional enzyme complexes in barley
Source: Front Plant Sci. 2025 Oct 16;16:1671162. doi: 10.3389/fpls.2025.1671162 (PMC12571849; doi:10.3389/fpls.2025.1671162)
Supplement: Supplementary file 3 [file Table1.docx]

Supplementary Table 1: Gene ID Information for Large and Small Subunits of AGPase in Different Crops

| Crop name | Gene name | Gene ID |
| --- | --- | --- |
| Barley | *HvAGPS1* | AAO16183 |
|  | *HvAGPS2a* | CAA88449 |
|  | *HvAGPS2b* | CAA88450 |
|  | *HvAGPL1* | CAA47626 |
|  | *HvAGPL2* | AAC49729 |
| Maize | *ZmAGPS1* | AY032604 |
|  | *ZmAGPS2a-1* | AF330035 |
|  | *ZmAGPS2a-2* | DQ118038 |
|  | *ZmAGPS2b* | AF334960 |
|  | *ZmAGPL1* | BT016868 |
|  | *ZmAGPL2* | Z38111 |
|  | *ZmAGPL3* | EF694838 |
|  | *ZmAGPL4* | EF694839 |
| Wheat | *TaAGPS1* | AY727927 |
|  | *TaAGPS2a* | X66 080 |
|  | *TaAGPS2b* | EU582678 |
|  | *TaAGPL1* | Z21969 |
|  | *TaAGPL2* | DQ406820 |
| Rice | *OsAGPS1* | AY028315 |
|  | *OsAGPS2a* | EF122437 |
|  | *OsAGPS2b* | AP004459 |
|  | *OsAGPL1* | AY028314 |
|  | *OsAGPL2* | D50317 |
|  | *OsAGPL3* | NM-001065811 |
|  | *OsAGPL4* | NM-001057719 |
