# Supplementary material for: Expression and interaction of AGPase subunits reveal functional enzyme complexes in barley
Source: Front Plant Sci. 2025 Oct 16;16:1671162. doi: 10.3389/fpls.2025.1671162 (PMC12571849; doi:10.3389/fpls.2025.1671162)
Supplement: Supplementary file 4 [file Table2.docx]

Supplementary Table 2: Information on Interacting Proteins Identified by IP-MS

| IP-MS | Gene ID | Protein Information | Coverage | Pepi-des | Note |
| --- | --- | --- | --- | --- | --- |
| AGPS1 | XP_044956112.1 | glucose-1-phosphate adenylyltransferase small subunit | 60.89 | 25 | AGPS2b |
|  | XP_044973892.1 | pyruvate, phosphate dikinase 2 | 71.25 | 53 | PPDK |
|  | XP_044974641.1 | 14-3-3-like protein B | 78.92 | 20 | 14-3-3 |
|  | XP_044984294.1 | glucose-1-phosphate adenylyltransferase large subunit 1 | 82.17 | 42 | AGPL1 |
|  | XP_044966283.1 | stearoyl- [acyl-carrier-protein] 9-desaturase 5, chloroplastic | 20.18 | 7 | ACP |
| AGPS2a | XP_044955733.1 | glyceraldehyde-3-phosphate dehydrogenase | 84.36 | 27 | GAPDH |
|  | XP_044973885.1 | pyrophosphate-energized vacuolar membrane proton pump-like | 26.87 | 21 | v-ppase |
|  | XP_044964740.1 | H/ACA ribonucleoprotein complex subunit 4-like | 20.48 | 8 | H/ACA |
|  | XP_044974641.1 | 14-3-3-like protein B | 78.9 | 20 | 14-3-3 |
|  | XP_044973892.1 | pyruvate, phosphate dikinase 2 | 71.64 | 53 | PPDK |
|  | XP_044954545.1 | NADP-dependent D-sorbitol -6-phosphate dehydrogenase-like | 43.59 | 11 | PDH |
|  | XP_044963005.1 | ATP synthase subunit beta, chloroplastic | 50.62 | 18 | ATP synthase |
|  | XP_044956112.1 | glucose-1-phosphate adenylyltransferase small subunit | 60.89 | 18 | AGPS2b |
| AGPS2b | XP_044973892.1 | pyruvate, phosphate dikinase 2 | 71.64 | 53 | PPDK |
|  | XP_044974641.1 | 14-3-3-like protein B | 78.9 | 20 | 14-3-3 |
|  | XP_044973885.1 | pyrophosphate-energized vacuolar membrane proton pump-like | 26.47 | 21 | v-ppase |
|  | XP_044946641.1 | glucose-1-phosphate adenyly-ltransferase small subunit 1 | 63.29 | 23 | AGPS1 |
|  | XP_044984294.1 | glucose-1-phosphate adenylyltransferase large subunit 1 | 82.79 | 42 | AGPL1 |
|  | XP_044974394.1 | Glutamate decarboxylase-like isoform X1 | 43.18 | 18 | GAD |

**Coverage: Peptide coverage percentage; Pepi-des: Number of peptide segments.**
