# Supplementary material for: Expression and interaction of AGPase subunits reveal functional enzyme complexes in barley
Source: Front Plant Sci. 2025 Oct 16;16:1671162. doi: 10.3389/fpls.2025.1671162 (PMC12571849; doi:10.3389/fpls.2025.1671162)
Supplement: Supplementary file 5 [file Table3.docx]

Supplementary Table 3: Primer Sequences for AGPase Gene Research

| Gene | Primer sequence |
| --- | --- |
| *HvAGPS1*-F | ATGGCGATGGCGATGGCGACGG |
| *HvAGPS1*-R | CTATATGACTGTCCCGCTAGGA |
| *HvAGPS2a*-F | ATGGATGTACCTTTGGCATCTA |
| *HvAGPS2a*-R | TCATATGACTGTTCCACTAGGG |
| *HvAGPS2b*-F | ATGGCGATGGCCGCGGCCGCCT |
| *HvAGPS2b*-R | TCATATGACTGTTCCACTAGGG |
| *HvAGPL1*-F | ATGTCATCGATGCAGTTCAGCA |
| *HvAGPL1*-R | CTACACGACGGTGCCGTCCTTG |
| *HvAGPL2*-F | ATGGACCTGCGCGTCGCTGCCC |
| *HvAGPL2*-R | TCATATGACTAATCCATCCGCGA |
| qPCR-*HvAGPS1*-F | TTATGGCTGTTTGAGGAA |
| qPCR-*HvAGPS1*-R | GCCCAGTATGGTGGTGTC |
| qPCR-*HvAGPS2a*-F | AAAGGAGAACAGTTGAAA |
| qPCR-*HvAGPS2a*-R | CAGTAACCGTCGTATAGG |
| qPCR-*HvAGPS2b*-F | AAAGGAGAACAGTTGAAA |
| qPCR-*HvAGPS2b*-R | CAGTAACCGTCGTATAGG |
| qPCR-*HvAGPL1*-F | CAATCGCCACATTCACCG |
| qPCR-*HvAGPL1*-R | AGTCCTCAAGCACCCAGA |
| qPCR-*HvAGPL2*-F | GTATTGATTCTTTCGGGC |
| qPCR-*HvAGPL2-*R | TCTTTTCTGCTTCCTCCT |
| 30a-*HvAGPS1*-F | GACAGCCCAGATCTGGGTAC  CATGGCGATGGCGATGGCG |
| 30a-*HvAGPS1*-R | TTGTCGACGGAGCTCGAATTCCT  ATATGACTGTCCCGCTAGGAATT |
| 30a-*HvAGPS2a*-F | gccatggctgatatcggatccA  TGGATGTACCTTTGGCATCTAAA |
| 30a-*HvAGPS2a*-R | ttgtcgacggagctcgaattcTCA  TATGACTGTTCCACTAGGGAGTAA |
| 30a-*HvAGPS2b*-F | GACAGCCCAGATCTGGGTA  CCATGGCGATGGCCGCGGCC |
| 30a-*HvAGPS2b*-R | TTGTCGACGGAGCTCGAATTCTCA  TATGACTGTTCCACTAGGGAGTAA |
| 30a-*HvAGPL1*-R | TTGTCGACGGAGCTCGAATT  CCTACACGACGGTGCCGTCC |

**Continued**

| 4T-*HvAGPL2*-F | gatctggttccgcgtggat  ccATGGACCTGCGCGTCGCT |
| --- | --- |
| 4T-*HvAGPL2*-R | ctcgagtcgacccgggaattc  TCATATGACTAATCCATCCGCG |
| AD-*HvAGPS1*-F | GCCATGGAGGCCAGTGAAT  TCATGGCGATGGCGATGGCG |
| AD-*HvAGPS1*-R | CAGCTCGAGCTCGATGGATCCCT  ATATGACTGTCCCGCTAGGAATT |
| AD-*HvAGPS2a*-F | GCCATGGAGGCCAGTGAATTCA  TGGATGTACCTTTGGCATCTAAA |
| AD-*HvAGPS2a*-R | CAGCTCGAGCTCGATGGATCCTCA  TATGACTGTTCCACTAGGGAGTAA |
| AD-*HvAGPS2b*-F | GCCATGGAGGCCAGTGAAT  TCATGGCGATGGCCGCGGCC |
| AD-*HvAGPS2b*-R | CAGCTCGAGCTCGATGGATCCTCA  TATGACTGTTCCACTAGGGAGTAA |
| BK-*HvAGPL1*-F | ATGGCCATGGAGGCCGAATTC  ATGTCATCGATGCAGTTCAGCA |
| BK-*HvAGPL1*-R | CCGCTGCAGGTCGACGGATC  CCTACACGACGGTGCCGTCC |
| BK-*HvAGPL2*-F | ATGGCCATGGAGGCCGAAT  TCATGGACCTGCGCGTCGCT |
| BK-*HvAGPL2-*R | CCGCTGCAGGTCGACGGATCC  TCATATGACTAATCCATCCGCG |
