# Supplementary material for: Expression and interaction of AGPase subunits reveal functional enzyme complexes in barley
Source: Front Plant Sci. 2025 Oct 16;16:1671162. doi: 10.3389/fpls.2025.1671162 (PMC12571849; doi:10.3389/fpls.2025.1671162)
Supplement: Supplementary file 6 [file DataSheet1.zip › 1/CoIP-MS/20240813003.xiboai-LC-MSMS90.zip!/微纳菲-定性质谱peaks online软件搜库结果查看说明.docx]

**PEAKS Online结果查看说明**

目录

[一、结果文件说明	1](#_Toc2172)

[二、表格文件的查看说明	2](#_Toc14895)

[2.1 表格文件信息	2](#_Toc31422)

[2.2 proteins文件基本信息	2](#_Toc19854)

[2.3 protein-peptide文件基本信息	3](#_Toc5977)

[三、数据处理与差异分析	4](#_Toc13364)

一、结果文件说明

打开报告文件夹，会有4个Excel（WPS）表格可打开的csv文件如下：

表格包括基于数据库检索得到的（db）protein-peptides、proteins表格，基于db搜库结果，满足一定一级定量值强度蛋白才会进行非标记定量(lfq)，产生对应的protein-peptides、proteins表格。

# 二、表格文件的查看说明

## 2.1 表格文件信息

表1： 两个csv文件详细信息

| **protein-peptides** | 鉴定出的蛋白所对应肽段信息 |
| --- | --- |
| **proteins** | 质谱鉴定到的蛋白列表 |

## 2.2 proteins文件基本信息

表2： protein结果文件中关键字说明

| **Protein Group** | 功能、序列等相近的蛋白可能有相近的肽段序列，有相同序列的蛋白会归总为一个Protein Group，使用一个序号 |
| --- | --- |
| **Top** | 一般只使用Protein Group中覆盖度最高的的主蛋白，Top蛋白筛选为TRUE进行数据处理，也可检查关注的蛋白 |
| **Accession** | 数据库中蛋白对应的ID号，是不同蛋白特征的编号。 |
| **-10lgP** | 蛋白可信度得分，越高越可信，20对应P值0.01 |
| **Coverage (%)** | 鉴定到的肽段在蛋白上的覆盖度，一般越高表示蛋白可信度越高。 |
| **Coverage Sample(%)** | 各样品鉴定到的肽段在蛋白上的覆盖度，一般相对值越高表示蛋白相对丰度越高。 |
| **Area** | 蛋白在各样品中的一级峰面积，可用于不同样品间的定量比较（较粗略）。 |
| **#Peptides** | 鉴定蛋白包含的肽段数量 |
| **#Unique** | 鉴定蛋白包含的特征性肽段数量 |
| **#Spec** | 蛋白在各样品中的肽段匹配到二级谱图的数量 |
| **PTM** | 蛋白鉴定到的修饰类型 |
| **Avg. Mass** | 蛋白平均分子量 |
| **Description** | 数据库中蛋白质的信息 |

**注：**

**1、Accession：**数据库中蛋白对应的ID号，是不同蛋白特征的编号。搜库结果中显示的Accession号一般是J9TAY5∣J9TAY5_IPOB或者sp∣P10965∣SPORB_IPOBA格式。则客户可用”∣”前面的编号J9TAY5或者“sp”和“∣”之间的P10965在uniprot（http://www.uniprot.org/uniprot）网站上搜索此蛋白编号对应具体蛋白、基因、氨基酸序列等信息。

**3、Description：**蛋白名称的详细描述；例如，对P10809蛋白编号的60kDa heat shock protein,mitochondrial OS=Homo sapiens GN=HSPD1 PE=1 SV=2，其中60kDa heat shock protein,mitochondrial是蛋白名称；OS=Homo sapiens是物种的拉丁文名称；GN表示gene name，基因名称；PE是ProteinExistence，即蛋白的可靠性，分5个等级，数据越小代表可靠性越高；SV：SequenceVersion，蛋白序列版本。

## 2.3 protein-peptide文件基本信息

表3 protein **-**peptide结果文件中关键字说明

| **Protein Group** | 功能、序列等相近的蛋白所在组，一般只展示某个组的主蛋白，即Top蛋白为TRUE |
| --- | --- |
| **Accession** | 数据库中蛋白对应的ID号 |
| **Peptide** | 肽段序列 |
| **Unique** | 是否是特异性肽段（Y=Yes、N=NO） |
| **-10lgP** | 该肽段对应图谱鉴定的可信度 |
| **Mass** | 此肽段的分子量 |
| **Length** | 此肽段氨基酸数目 |
| **ppm** | 此肽段质量与理论值偏差 |
| **m/z** | 该肽段质荷比 |
| **z** | 电荷 |
| **RT** | 保留时间，以min为单位 |
| **Area** | 肽段在不同样品中的强度 |
| **Scan** | 质谱扫描到该肽段的编号 |
| **Source File** | 文件来源，肽段对应的样品名称 |
| **#Spec** | 该肽段匹配谱图数 |
| **Start-End** | 肽段在蛋白中的起始和终止的位置 |
| **PTM** | 肽段修饰类型 |
| **AScore** | 用于计算修饰位点可信度 |

可以根据此表，查看感兴趣的蛋白对应的肽段序列、唯一肽段序列。在文章发表过程中会添加目标蛋白对应肽段的二级谱图，一般质谱图上也会出现保留时间和质荷比信息。

注：一般来说，想了解鉴定到的蛋白的信息，只参考proteins表格即可，想了解蛋白与肽段的关系，需要参考protein-peptide表格。

# 三、数据处理与差异分析

Lfq.protein.csv表格中的Area值是用来进行蛋白差异分析的，原始数据中Area会有缺失部分，为了更好的反应样本间的生物学差异，需要对原始数据进行筛选及优化。如没有重复组，需对缺失部分可赋予一个最小值（如最小值的1/5）。

已经优化的数据可用作分析差异蛋白，筛选条件包括差异倍数与统计检验值。可用实验组的Area与对照组的Area比值得到（Fold Change,FC），差异倍数一般设置为2倍（可根据实际情况选择1.2、1.5、2倍）。统计检验值可使用t-test计算所得，阈值设定为PValue<=0.05或PValue<=0.01，如果蛋白同时满足条件（FC >= 2或 FC <= 0.5,PValue <= 0.05或 PValue <= 0.01）则老师可将该蛋白作为差异表达蛋白。

Peaks 自带Sample Profile (Ratio)以及F检验（F-test）的Significance结果，但请注意，对于不重复组（独立样本），F检验可以用于比较各组的均值是否存在显著性差异。这在统计学的一般假设检验框架下是有效的，前提是满足一些基本假设，包括：正态性假设即每个组的观测值应该来自于一个正态分布以及方差齐性假设：不同组的方差应该是相等的。当只有两组时，F检验与独立样本的t检验等价。当有多个组时，F检验可以用于比较全体组的均值，但如果F检验显示存在显著性差异，通常需要进行进一步的事后分析（post hoc analysis）来确定哪些组之间存在显著差异，老师可自行评估使用。
