# Supplementary material for: Expression and interaction of AGPase subunits reveal functional enzyme complexes in barley
Source: Front Plant Sci. 2025 Oct 16;16:1671162. doi: 10.3389/fpls.2025.1671162 (PMC12571849; doi:10.3389/fpls.2025.1671162)
Supplement: Supplementary file 6 [file DataSheet1.zip › 1/CoIP-MS/20240813003.xiboai-LC-MSMS90.zip!/蛋白质溶液质谱检测报告.docx]

**LC-MS/MS质谱蛋白质检测分析报告**

**项目名称：	蛋白溶液质谱分析**

**项目编号：	20240813003**

**委托单位：	四川农业大学**

**项目联系人：	席博爱**

**报告日期：	2024 年	8 月 26 日**

检测单位：深圳市微纳菲生物技术有限公司

**目录**

**[LC-MS/MS质谱蛋白质检测分析报告	0](#_Toc22261)**

**[1. 样品信息	2](#_Toc4901)**

**[2. 实验材料和方法	2](#_Toc26243)**

[2.1. 实验材料	2](#_Toc23307)

[2.2. 样品前处理	3](#_Toc24122)

**[3. LC-MS/MS检测与数据库检索分析	4](#_Toc27724)**

[3.1. LC-MS/MS检测	4](#_Toc3433)

[3.2. 数据库检索分析	4](#_Toc20871)

**[4. 实验结果	5](#_Toc12926)**

**[5. 公司简介	6](#_Toc23703)**

1. **样品信息**

| **样品名称** | **上机编号** | **样品量** | **溶液成分** |
| --- | --- | --- | --- |
| AGS1 | 0813003-1 |  |  |
| AGPS2a | 0813003-2 |  |  |
| AGPS2b | 0813003-3 |  |  |
| ck | 0813003-4 |  |  |

2. **实验材料和方法**
   2.1. **实验材料**
        2.1.1. **主要试剂和耗材**

| **试剂或耗材** | **品牌** |
| --- | --- |
| 尿素 Urea | Sigma |
| 碳酸氢铵 Ammonium bicarbonate | Sigma |
| 二硫苏糖醇 Dithiothreitol | Sigma |
| 碘乙酰胺 Iodoacetamide | Sigma |
| Trypsin蛋白酶 | 深圳市微纳菲生物技术有限公司（Wininnovte Bio） |
| 乙腈Acetonitrile | Merck |
| 甲酸Formic acid | Thermo fisher |
| 超滤离心管（Nanosep Centrifugal Devices 10 kD） | Pall |

2.1.2. **主要仪器**

| **仪器** | **型号** | **品牌** |
| --- | --- | --- |
| 冷冻离心机 | Fresco 17 | Thermo Scientific |
| 真空冷冻干燥机 | Savant DNA120 | Thermo Scientific |
| 纳升液相系统 | Easy-nLC1200 | Thermo Scientific |
| 高分辨质谱仪 | Orbitrap Eclipse | Thermo Scientific |

2.2. **样品前处理**

蛋白酶解方法参考FASP[Jacek R Wisniewski, Alexandre Zougman, Nagarjuna Nagaraj and Matthias Mann. Nature Methods, 2009, 6:359 - 362]，具体如下：

1) 将蛋白溶液样品转移至对应的10KD的超滤管中，12,000 g离心20min；
2) 每个超滤管各加200 μl Buffer1（8M urea/100 mM Tris-HCl，pH 8.5），充分溶解变性蛋白；
3) 加入20 μl DTT（100mM）溶液，37℃反应2 h还原二硫键；
4) 加入20μl IAA（500 mM）溶液，室温避光反应15 min；
5) 将还原烷基化后的蛋白溶液12,000 g离心20 min，弃掉收集管底部溶液。加入200uL Buffer1 离心，重复2次；
6) 加200 μl Buffer2（8M urea/100 mM Tris-HCl，pH 8.0），12,000 g离心20 min，弃掉收集管底部溶液，重复1次；
7) 加200 μl 碳酸氢铵溶液（25 mM），12,000 g离心20 min，弃掉收集管底部溶液，重复2次；
8) 更换新收集管，加入100 μl胰酶溶液（0.01ug/ul），37℃反应14 h；
9) 取出超滤管，12,000 g离心20 min，收集酶解后肽段；
10) 超滤管中再加入100 μl 碳酸氢铵溶液（25 mM），12,000 g离心10 min，收集管底溶液并与前次溶液合并；
11) 离心干燥，待做上机。

Aliquots of proteins were mixed with 200 ul of 8M urea in Nanosep Centrifugal Devices (PALL). The device was centrifuged at 12,000g at 20℃ for 20 min. All following centrifugation steps were performed applying the same conditions allowing maximal concentration. Then, 200 μl of 8M urea solution with 10 mM DTT were added, and the reduction reaction was kept for 2 h at 37 °C. The solution was removed by centrifugation, and 200ul 8M urea solution with 50 mM iodoacetamide (IAA) was added. The sample was incubated in the dark for 15 min at room temperature. The ultra-fraction tube was washed with 200 μl of 8 M urea three times and 200 μl of 25 mM ammonium bicarbonate three times by centrifugation at 12,000g for 20 min at room temperature. Then, 100 μl of 25 mM ammonium bicarbonate containing 0.01 μg/μl trypsin was added to each filter tube. The tubes were incubated at 37 °C for 12 h. The filter tubes were washed twice with 100 μl of 25 mM ammonium bicarbonate by centrifugationat 12,000g for 10 min. The flow-through fractions were collected and lyophilized.

3. **LC-MS/MS检测与数据库检索分析**
   3.1. **LC-MS/MS检测**

酶切多肽样品经离心干燥后，重新溶解于Nano-LC 流动相A（0.1%甲酸/水）中装瓶上样，进行在线LCMS分析。溶解后的样品以合适的体积上样到nanoViper C18预柱上（3μm，100Å），然后20ul体积冲洗脱盐。液相为Easy-nLC1200纳升液相系统（ThermoFisher, USA），样品在预柱上脱盐保留后再经分析柱分离，分析柱规格是C18反相色谱柱（Acclaim PepMap RSLC,75 μm×25 cm C18-2 μm 100 Å），实验所用梯度为90 min内流动相B（80%乙腈，0.1%甲酸）由5%升高至40%。质谱采用ThermoFisher Orbitrap Eclipse系统（ThermoFisher, USA）结合纳升喷雾Nano Flex离子源（ThermoFisher, USA），喷雾电压为2kV，离子传输管加热温度为320℃。质谱扫描方式为信息依赖的采集工作模式下（DDA，Data Dependent Analysis），一级质谱扫描分辨率为60000，扫描范围350-1500m/z，最大注入时间118ms。每次DDA循环时间为2s，二级质谱离子最大注入时间为22ms，碰撞室能量（高能碰撞诱导解离, HCD）设定为30%，适用于所有前体离子，动态排除设置为60秒。

The peptide samples are reconstituted in Nano-LC mobile phase A (0.1% formic acid/water) and loaded into vials for online LCMS analysis. The reconstituted samples are loaded into a nanoViper C18 pre-column (3μm, 100Å) with an appropriate volume, followed by a 20μL volume wash for salt removal. The liquid chromatography system used is the Easy-nLC1200 nano HPLC system (ThermoFisher, USA), where samples are desalted and retained on the pre-column before being separated on the analytical column. The specifications of the analytical column are a C18 reverse-phase chromatography column (Acclaim PepMap RSLC, 75 μm×25 cm C18-2 μm 100 Å), and the gradient used in the experiment increases the percentage of mobile phase B (80% acetonitrile, 0.1% formic acid) from 5% to 40% over a period of 90 minutes.The mass spectrometry is performed using the ThermoFisher Orbitrap Eclipse system (ThermoFisher, USA) coupled with a nano spray Nano Flex ion source (ThermoFisher, USA), with a spray voltage of 2kV and a heated ion transfer tube temperature of 320°C. The mass spectrometry scan mode is set to Data Dependent Analysis (DDA), where the resolution of the MS1 scan is 60,000 with a scan range of 350-1500 m/z and a maximum injection time of 118 ms. Each DDA cycle time is set to 2 seconds, and the maximum injection time for the MS2 ions is 22 ms. The collision cell energy (HCD, High Energy Collision-Induced Dissociation) is set at 30%, applicable to all precursor ions, with a dynamic exclusion set to 60 seconds.

3.2. **数据库检索分析**

质谱采集的原始raw数据文件，采用PEAKS Online 11 (Bioinformatics Solutions Inc.Waterloo, Canada)软件进行加工处理和检索分析。数据库为提供的目的蛋白序列，检索参数设置如下：胰蛋白酶酶解，一级质谱质量容差为10ppm，二级质谱为0.05Da，酶切割最大漏切数为4；固定修饰：半胱氨酸碘乙酰胺化；可变修饰：蛋白质N端乙酰化（Acetylation(Protein N-term)），天冬酰胺/谷氨酰胺脱氨化（Deamidation (NQ)），甲硫氨酸氧化（Oxidation (M)），谷氨酸焦谷氨酸化（Pyro-glu），谷氨酰胺焦谷氨酰环化（Pyro-Gln)。

The MS/MS data were analyzed for protein identiﬁcation and quantiﬁcation using PEAKS Online 11.The local false discovery rate at PSM was 1.0% after searching against target protein sequence database with a maximum of four missed cleavages.The following settings were selected: Oxidation (M), Acetylation (Protein N-term), Deamidation (NQ), Pyro-glu from E, Pyro-glu from Q for variable modifications as well as fixed Carbamidomethylation of cysteine. Precursor and fragment mass tolerance were set to 10 ppm and 0.05 Da.

4. **实验结果**

具体见相关excel表格。

5. **公司简介**

深圳市微纳菲生物技术有限公司是一家专注于蛋白质组学、代谢组学及生物信息学分析的国家高新技术企业，主要开展质谱组学技术服务和解决方案。提供相关核心试剂和耗材。主要核心业务有：（1）**技术服务：**蛋白鉴定，DDA, DIA, 4D定量蛋白质组学，修饰蛋白质组学，抗体/重组蛋白全长测序，代谢/脂质组学；**（2）产品：**质谱级胰酶，Lys-C酶，Glu-C酶，糖苷酶F；微量质谱上样V底瓶；各种规格SPE多肽除盐柱；**（3）代理:** 海能悟空液相色谱仪器K2025.

微信公众号

电话：0755-86707180

邮箱：wnf@wininnovatebio.com

网址：<http://www.wininnovatebio.com>

业务经理：方经理，15818789168；王经理：13590154171

地 址：深圳市南山区桃源街道丽山路10号大学城创业园401室
